# Ten Simple Rules for Cultivating Open Science and Collaborative R&D

**DOI:** 10.1371/journal.pcbi.1003244

**Published:** 2013-09-26

**Authors:** Hassan Masum, Aarthi Rao, Benjamin M. Good, Matthew H. Todd, Aled M. Edwards, Leslie Chan, Barry A. Bunin, Andrew I. Su, Zakir Thomas, Philip E. Bourne

**Affiliations:** 1 Waterloo Institute for Complexity and Innovation, Waterloo, Ontario, Canada; 2 Results for Development Institute, Washington, D.C., United States of America; 3 Department of Molecular and Experimental Medicine, Scripps Research Institute, La Jolla, California, United States of America; 4 School of Chemistry, University of Sydney, Sydney, New South Wales, Australia; 5 Structural Genomics Consortium, University of Toronto, Toronto, Ontario, Canada; 6 Department of Social Sciences, University of Toronto Scarborough, Toronto, Ontario, Canada; 7 Collaborative Drug Discovery, Burlingame, California, United States of America; 8 Council of Scientific and Industrial Research, New Delhi, India; 9 Skaggs School of Pharmacy and Pharmaceutical Sciences, University of California San Diego, La Jolla, California, United States of America; Whitehead Institute, United States of America

How can we address the complexity and cost of applying science to societal challenges?

Open science and collaborative R&D may help [1]–[3]. *Open science* has been described as “a research accelerator” [4]. Open science implies open access [5] but goes beyond it: “Imagine a connected online web of scientific knowledge that integrates and connects data, computer code, chains of scientific reasoning, descriptions of open problems, and beyond …. tightly integrated with a scientific social web that directs scientists' attention where it is most valuable, releasing enormous collaborative potential.” [1].

Open science and collaborative approaches are often described as *open source*, by analogy with open-source software such as the operating system Linux which powers Google and Amazon—collaboratively created software which is free to use and adapt, and popular for Internet infrastructure and scientific research [6], [7]. However, this use of “open source” is unclear. Some people use “open source” when a project's results are free to use, others when a project's process is highly collaborative [4].

It is clearer to classify open source and open science within a broader class of *collaborative R&D*, which can be defined as scalable collaboration (usually enabled by information technology) across organizational boundaries to solve R&D challenges [8].

Many approaches to open science and collaborative R&D have been tried [1], [9]. The Gene Wiki has created over 10,000 Wikipedia articles, and aims to provide one for every notable human gene [10]. The crowdsourcing platform InnoCentive has reportedly facilitated solutions to roughly half of the thousands of technical problems posed on the site, including many in life sciences such as the $1 million ALS Biomarker Prize [11]. Other examples include prizes (X-Prize [12]), scientific games (FoldIt [13]), and licensing schemes inspired by open-source software (BIOS [14]).

Collaborative R&D approaches vary in openness [15]. In some approaches, the R&D process and outputs are open to all—for example, open-science projects like the Gene Wiki described above. In other approaches which demonstrate what might be called *controlled collaboration*, there are strong controls on who contributes and benefits—for example, computational platforms like Collaborative Drug Discovery or InnoCentive that support both commercial and nonprofit research [9], [11].

Collaborative approaches can unleash innovation from unforeseen sources, as with crowdsourcing health technologies [11]–[13], [16]. They may help in global challenges like drug development [17], as with India's OSDD (Open Source Drug Discovery) project that recruited over 7,000 volunteers [16] and an open-source drug synthesis project that improved an existing drug without increasing its cost [18].

If you want to apply open science and collaborative R&D, what principles are useful? We suggest Ten Simple Rules for Cultivating Open Science and Collaborative R&D. We also offer eight conversational interviews exploring life experiences that led to these rules (Box 1).

**Box 1. Conversations on Open Science and Collaborative R&D**

Many commentators have considered challenges in translating open science and collaborative methods to biomedical research [2]–[4], [9], [17], [20], [24], [26], [28], [29]. How can protecting intellectual property be balanced with freeing researchers to build on previous knowledge? If R&D results are collaboratively created and freely available, who will take responsibility for costly clinical trials and quality control? What will be the Linux of open-source R&D?

To explore such challenges and convey life experiences in biomedical open science and collaborative R&D, we offer eight conversational interviews by the first author of this article as supplementary material. The conversations were done on behalf of the Results for Development Institute and are with:

- **Alph Bingham**, cofounder of InnoCentive (Text S1)
- **Barry Bunin**, CEO of Collaborative Drug Discovery (Text S2)
- **Leslie Chan**, open access pioneer and director of Bioline International (Text S3)
- **Aled Edwards**, director of the Structural Genomics Consortium (Text S4)
- **Benjamin Good**, coleader of the Gene Wiki initiative (Text S5)
- **Bernard Munos**, pharmaceutical innovation thought leader (Text S6)
- **Zakir Thomas**, director of India's Open Source Drug Discovery (OSDD) project (Text S7)
- **Matt Todd**, open science and drug development pioneer (Text S8)

## Rule 1: Get the Incentives Right—Learn from the Past

Why should contributors take part in your project? Learn from incentives that have worked in mass collaborations and open-source software, such as reputation building, enjoyment, cooperatively solving interesting problems that are too hard to do alone, and jointly developing tools that benefit all developers [6], [7], [19]. Organizational incentives can include lowering costs, tapping external innovation, implementing novel business models such as selling complementary services, and jointly competing for public admiration or grant funding. Altruism can motivate collaboration, but frequently it is not the main reason [9]. With this in mind, align individual incentives with collective benefit [1]. Look to past and present precompetitive collaborations for ways to address intellectual property and competitive concerns [3]. Share attribution with contributors so they can advance their goals and demonstrate their capabilities.

## Rule 2: Make Your Controlled Collaborations Win-Win-Win

Perhaps completely open science seems unsuitable to you, if for example you are engaged in market-driven R&D that must recoup investments. There are ways to benefit from open science and collaborative methods while retaining appropriate controls and the opportunity to provide public benefit. You, your partners, and the public can all benefit—a win-win-win situation. You might use computational platforms to supercharge information sharing with selected partners, including public-benefit initiatives that match your mission [9]. You might use crowdsourcing to overcome roadblocks by opening up chosen parts of your R&D process to new innovators [11]. Or you might make public selected data or software tools, exporting them to the open-source realm to gain from goodwill or quality improvement [3]. Sharing can make both business and social sense, whether in implementing open standards, collaborating precompetitively, or reducing duplication of effort [20]. Keep an eye open for opportunities to “do well by doing good” by structuring initiatives for private and public benefit [21]. Collaborative approaches can benefit both public and private sectors in collaborating across competitive boundaries, connecting problems with problem solvers, and cultivating a knowledge commons [1], [9].

## Rule 3: Understand What Works—and What Doesn't

You can save yourself frustration by not using an unsuitable collaborative method, be it a Wiki without an audience or a crowdsourced research challenge without focus [8]. Consider questions like: have you learned from others who have tried the method? Do you understand when the method fails, and what is necessary for it to work? Is there a good match between the method and your goals? Are you contributing your experiences and interesting failures back to the community, thus demonstrating thought leadership? If you are interested in more effective knowledge sharing, consider low-budget opportunities such as starting an online Q&A site about open science or collaborative R&D using a platform like StackExchange. There are also opportunities to help evaluate what really works—moving beyond anecdotal evidence to case studies and metrics.

## Rule 4: Lead as a Coach, Not a CEO

The command-and-control style doesn't work well with contributors from diverse organizations, many of whom may be volunteers [22]. And as has been said of Linus Torvalds, the founder of the open-source operating system Linux, “Linus doesn't scale”: leaders of mass collaborations can become bottlenecks unless they encourage distributed workflows and leadership [7]. Be flexible about management (but strict about quality). Check your ego at the door—you're playing a team game and will be stronger when others want to contribute. Participants will feel more motivated if their contribution enriches a joint resource rather than just the leader. Can you give up exclusive ownership and credit to achieve with others what you cannot achieve alone?

## Rule 5: Diversify Your Contributors

A powerful aspect of collaborative R&D is the potential diversity of the community—including students [16], patients [23], gamers [13], and researchers from lesser-known countries or institutions. You can use open science to attract diverse contributors by lowering barriers to participation, publicly tackling audacious challenges (see Rule 8), and making collaboration fun. Consider open licensing terms and joint or public ownership of selected outcomes to broaden your participant base [14], [15], [21], [24]. Encourage all community members to find ways to contribute that suit their abilities and inclinations. Can you reach past your usual partners, and make it easy for others to get up to speed with what you're doing? Are there opportunities for “citizen science,” perhaps through organizing many microcontributions [1], [13]?

## Rule 6: Diversify Your Customers

Can you engage the broadest possible base as beneficiaries? The science that you do in the open spreads its benefits widely, and that can attract unexpected accolades and collaborators [1], [4]. Productively involving stakeholders can inform your research—for example, through participatory research strategies involving the people your efforts are meant to help [25]. Contributing to collaborative initiatives targeting human development challenges can motivate your team, and potentially lead to innovations that are transferable to for-profit markets. Neglected disease R&D is a case in point which seems particularly suitable for collaborative pilot projects, given its lower profits, humanitarian appeal, and need for new methods [26]. If your work is commercially driven, consider humanitarian licensing approaches that encourage nonprofit applications by others to poorer demographics [2], [21].

## Rule 7: Don't Reinvent the Wheel

The more you can use what already exists, the greater your effectiveness will be. Are there lab and computational resources that could be used when otherwise idle? Can you find people already working on elements of your problem, and organize their collective work? Before starting a new initiative, have you explored and considered joining existing ones? Piggybacking on active efforts eases prototyping and gathering enthusiastic initial users. Build on the cumulative stockpile of past open initiatives (see Rules 1 and 3).

## Rule 8: Think Big

For projects hoping to harness the power of mass collaboration, a major challenge can be attracting a large community of contributors. Many of the best mass collaborations orient around seemingly audacious goals like: “build a free encyclopedia of all the world's knowledge” (Wikipedia), “develop a review article for every human gene” (Gene Wiki), and “build a new operating system” (Linux). Establishing a driving, high-level purpose will help spread the idea of your project and motivate people to come have a look and see what they can do. Be ready to scale with success.

## Rule 9: Encourage Supportive Policies and Tools

Can you cultivate open science and collaborative R&D by helping to make them part of “standard operating procedure”? For example, can you encourage institutional data sharing [24]? Can you build a profiling platform of collaborative initiatives, summarizing what they have achieved and what types of collaborators they are seeking? Do you have opportunities to adopt appropriate policies in your own organization or field? A case study to learn from is the spread of open access from wishful thinking to widespread fact [5].

## Rule 10: Grow the Commons

As intellectual property debates illustrate, there are legitimate differences of opinion on how best to motivate innovators' investments to generate new knowledge [21], [26]. But in the long run, sharing more knowledge and tools boosts both for-profit and nonprofit research [2], [3]. This growing shared resource of knowledge and tools—“the commons”—is the product of centuries of striving. It depends on cumulative win-win-win collaborations spanning organizations, nations, and generations. Can you find ways to advance your interests while remaining part of this larger narrative [1], [5], [19], [27]?

## Supporting Information

Text S1 A conversation with Alph Bingham, cofounder of InnoCentive.
(PDF)

Text S2 A conversation with Barry Bunin, CEO of Collaborative Drug Discovery.
(PDF)

Text S3 A conversation with Leslie Chan, open access pioneer and director of Bioline International.
(PDF)

Text S4 A conversation with Aled Edwards, director of the Structural Genomics Consortium.
(PDF)

Text S5 A conversation with Benjamin Good, coleader of the Gene Wiki initiative.
(PDF)

Text S6 A conversation with Bernard Munos, pharmaceutical innovation thought leader.
(PDF)

Text S7 A conversation with Zakir Thomas, director of India's Open Source Drug Discovery (OSDD) project.
(PDF)

Text S8 A conversation with Matt Todd, open science and drug development pioneer.
(PDF)
