# Supplementary material for: Ten Simple Rules for Cultivating Open Science and Collaborative R&D
Source: PLoS Comput Biol. 2013 Sep 26;9(9):e1003244. doi: 10.1371/journal.pcbi.1003244 (PMC3784487; doi:10.1371/journal.pcbi.1003244)
Supplement: Text S1 — A conversation with Alph Bingham, cofounder of InnoCentive. (PDF) [file pcbi.1003244.s001.pdf]

Insider Views of Collaborative R&D for Health: Q&A with Alph Bingham

August 23, 2012

Alph Bingham is the co-founder of Innocentive*, an online platform for crowdsourcing technical solutions. He is also the co-author of The Open Innovation Marketplace.*

HASSAN: *Alph, what's the big problem that InnoCentive is trying to solve?*

ALPH: Well, I guess the big problem was a realization that I had 30 years ago when I started doing R&D for a major corporation. It was the appreciation that the research problems that I was responsible for were generally exposed only to me, and a handful of individuals that I in turn chose to discuss them with.

And yet my prior experience in an academic environment was that problems would often be debated by 20 or 30 classmates. What always impressed me was the way in which the 20 or 30 classmates each solved the problems uniquely–based on their own personal experiences, the idiosyncrasies of their training, their prior research projects. Suddenly I was being called upon to solve problems, and I was appreciating the fact that there were about 29 perspectives missing.

InnoCentive was built to develop a mechanism for re-injecting all of those different perspectives into problem solving. After we had done that, we made discoveries about how problems are solved when they're widely exposed and where solutions come from–and, at times, the *infrequency* with which the best solution originates from somebody that would be, on paper, declared an expert in that particular challenge or problem.

HASSAN: *We're going to dive into that in a minute. First, although I think almost all of our readers will know about InnoCentive, it would be interesting to hear in your own words about InnoCentive in a nutshell.*

ALPH: InnoCentive looks to traditional R&D like bounty hunting looks to traditional law enforcement.

In the traditional case, you've got a prescribed body of problem solvers. The outlaws, whether in the form of a scientific challenge or in the form of an actual outlaw, present themselves and then that established body–R&D staff or law officers–tackles the challenge.

In the bounty hunting system, you determine the value of bringing in the outlaw in advance. Then you open it up and allow a large community–the entire world in an ideal situation–to self-select as volunteering to work on that particular problem, outlaw, whatever the case might be.

And so InnoCentive takes advantage of the Internet to allow it to basically post the outlaw's wanted poster on the world's largest marshal's office door.

HASSAN: *That's a great analogy. InnoCentive covers lots of fields, even if you limit your scope to problems relating to health. So following on your previous comment about frequencies or infrequencies of solutions, have you had higher success in particular types of challenges?*

ALPH: We actually define differing types of challenges, and success is often a function of the particular type.

By type what we generally refer to is, what are the rules of engagement? For example, an Ideation challenge may require that you only give top-of-mind solutions with no IP transfer. A Theoretical challenge may be the equivalent of asking you to write a white paper on the subject, and back your hypotheses up with literature precedents and grant non-exclusive rights of practice to the seeker. A Reduction to Practice challenge may require that you actually submit evidence that you have executed an approach to solving the problem that is successful and ultimately that will transfer IP rights to the seeker.

If I look at combined numbers for InnoCentive, overall problem solving success is about 40 percent. And maybe the Reduction to Practice challenges are on the 20 percent end of the range, and the Ideation challenges are on the 60 to 80 percent end of the range.

As far as appropriate challenges, the kinds that work well are the kinds for which you could describe a solution in advance. For example, without having any idea how to make drug X, I might be able to say that I want to make drug X in 98 percent yield at 99 percent purity and for a cost of less than $500 a kilo. Now that's a pretty good description of what a solution looks like, without telling you anything about the solution itself. Problems of that type work well because then the solvers know what they're shooting for. They tend to give more on-point submissions.

But that said, we're learning, through greater use of Ideation and Theoretical challenges, how to begin solving problems that fall into the category of "I'll know it when I see it." It's much harder to write a challenge in which there's a high degree of subjectivity to the solutions, but we're getting better at it.

HASSAN: *It's reminiscent of computational problems where finding a solution is hard but verifying a solution is easy. Following up on that, how would you characterize the types of health R&D challenges for which InnoCentive is* **not** *appropriate?*

ALPH: I think when the challenge can be articulated, with some criteria against which the solver can self-judge whether they're making progress–these are appropriate for distributing to a crowd. The crowd doesn't want to try to read your mind or guess what you're actually looking for.

You might be at the other end of the spectrum, which would be less appropriate for InnoCentive, in which you're saying, "Look, we know exactly what we want done. We know the one lab that's certified to do it. They've simply got to take a bunch of this stuff and put it in a humidity chamber and tell us how fast it decomposes." In that case, you probably want a contract lab with GMP certification to do it.

Now, you *can* prepend a mechanism like InnoCentive to have labs qualify themselves to engage with you. Perhaps it's not as straightforward as the example I just gave in which it's a standard ritualized recipe. Perhaps you're looking for somebody to help you develop new assay methodology for metal brittleness under a set of conditions in which standard methodology isn't satisfactory and for which there needs to be ready access to a company's ongoing materials for testing. In a case like that you can prepend a crowdsourcing engagement for ERFPs–electronic request for proposals–and then perhaps find partners that you wouldn't have found otherwise.

It's hard for me not to try to answer your question with "We can do that, we can do that, we can do that." But to be honest there is a state at which it's mundane recipe-following that's not appropriate for InnoCentive-type crowdsourcing, and there's a state of stumbling around that's not always appropriate for crowdsourcing. But it's a pretty rich middle.

HASSAN: *Fascinating. InnoCentive is in some sense the product of several decades of experience that*

you have had, along with a particular state of technology readiness: the Internet, mass collaborative tools, etc. If you push that forward five years, what fundamentally new things could come into platforms like InnoCentive that aren't there today?

ALPH: In terms of articulating a challenge, getting it exposed to a large number of minds and getting it solved, I don't see a lot of technological change. I joked earlier about the world's largest marshal's office door and yes, there were technological advances that made such a thing possible. We needed high bandwidth, we needed to be able to submit complex solutions, and if you called for a model of something, we needed enough commonality of software that I could run the model that you submitted.

Given that, I think that what has to happen now is perhaps as much social as technological. It's figuring out how these communities can help you with the broader part of the problem solving. For getting the problem stated correctly and framed right, we usually rely on InnoCentive's internal expertise plus our client's deep subject matter expertise. But ways in which the crowd can participate there are not thoroughly explored. I expect they will be.

And then there's the vetting of a variety of solutions. If I hand 10 novel ideas to you, you will probably be able to digest them, process them, and figure out which of the ones you're willing to bet on–even if they are a far cry from what you might have thought of in advance. But if I hand you 1000 of them, you're going to retreat to mechanisms like looking for familiarity, and then we're going to be reinserting expert opinion bias back into the process. So we've got to figure out how these communities can provide some of that vetting–so we can take 1000 ideas, boil them down to 10, and get you the right ones as the solution seeker.

HASSAN: *It sounds like it's inserting mass collaboration into the various parts of the process itself that's one key advance. I think I heard you start to hint that there's also a challenge of bringing open innovation into various parts of organizations themselves. In other words, open innovation of this sort only works if people within companies or within groups are open to using it, are culturally ready for it, and have the capacity to actually take outside solutions and bring them back within their organization.*

ALPH: That is absolutely true. In discussing open innovation, I had a colleague that used to describe it as, "the problem is gravity". As you're trying to escape from the pull of the central organization, you're fighting against it constantly.

If you do succeed in getting away and exploring open space away from that gravitational field, and find something interesting and want to bring it back–then gravity kicks in again, trying to ensure that you crash and burn upon re-entry.

HASSAN: *Organizational escape velocity.*

ALPH: Yes–this is one of the biggest boundaries to adoption.

Some kinds of open innovation have bigger cultural boundaries. For example, if you're talking about open innovation as collaborating with a university, scientists can readily agree to that notion. Most of your Ph.D. scientists went to a university. They liked that environment and resonated with it. So open innovation, if it means that we've got engagements with 25 major universities and our scientists meet and discuss ideas with them regularly, isn't a very culturally painful process.

On the other hand, if you take a crowdsourcing mechanism like InnoCentive in which you say, "Your problem's going to be broadcast. You're not going to engage with these solvers. They're going to submit to you solutions and then, without you knowing any of their credentials, we want you to tell us

which solution you consider to be the best fit against your criteria and the one that you want to issue an award on,"–that's going to be a bigger barrier. It may even turn out that the person with the best idea is somebody whose résumé you would have never given two looks at if they submitted it as a contractor or as a potential employee.

This even starts to threaten the underpinnings of the expertise that you owned and built up as an individual. It starts to get to your own identity as an expert problem solver. But it turns out that the taxpayers or the donors of the Gates Foundation or the shareholders of a company just want the problems solved, and don't care as much as you do about *who* solves them.

So the reality is that you need to rethink your role of "problem solver" as actually "solution finder," and you need to be somewhat indifferent to the source of that solution. I can tell you, that's an identity crisis for most of us scientists out there.

HASSAN: *Absolutely, and even though it sounds so simple when Henry Chesbrough talks about open innovation in his books, if you take it seriously, it requires a real role rethinking – almost a change of who you are.*

Alph, speaking of books, you recently co-authored The Open Innovation Marketplace. *What are one or two pieces of advice from that book for those who might be thinking of using open innovation, particularly for health R&D challenges?*

ALPH: Before I answer that question, I want to reinforce what you said. In fact, one of the paragraphs you'll find in that book effectively says that open innovation is a very simple practice to describe–but if you start to unpack it and its implications on the way you do business, it's really quite profound. I think that gets lost too easily because people want to take this open innovation idea and just bolt it onto the side of their organization.

Now I'm getting into some of the things that we say in the book that *are* advice. Once it's bolted onto their organization they basically want to keep doing things the way they've done them, but they want to add to that the merits of the crowd and of ideas flowing freely in. And they don't want to stop and realize that if they don't change some of the core of the way they do business, they will only dabble in open innovation. They will never reap the real benefits of it.

As an example, if they take a problem and they beat it to death internally before they open it up to novel solutions, and they get a novel solution and it solves the problem, that's great–but they also got those 15 internal failures that they've paid for along the way. To shift them to think about these non-inside channels at an appropriate or earlier time requires a rethinking of the way in which their R&D process is managed.

That's why I say the true virtues of open innovation are still not realized. In spite of all the good stories that I could tell, the real transformative value is not yet there.

HASSAN: *It sounds like you would advocate for not just the "fail fast" mentality that's familiar to many of us, but also a sort of "succeed fast" mentality where you might try these kinds of processes early on, see what happens, and then short-circuit some of the more laborious internal processes if the open innovation turns out well.*

ALPH: I would. There are a lot of processes I would turn upside down.

There are reasons for keeping work internal, like very high levels of confidentiality–but be careful if

you’ve been trained by your legal staff and others to just invoke that as a knee-jerk reaction. Think it through. What really is truly confidential, if it cannot be disclosed that you're even working on it, don’t disclose it. Don’t use open innovation–do that work inside. But I’ll tell you, it’s a fraction of what you think it is when you first think about it.

Sometimes you can’t afford to leave any stone unturned. If you crowdsourced it and you didn’t get ideas, you university-granted it and you didn’t get any good leads… then you might have to still tackle it internally, but those external failures weren’t failure at all. Some of the failed pathways you’ve allowed the market to take on your behalf–you don’t have to repeat. You can now take a combination of ideas that came from multiple innovation sources and stitch it together into something of commercial or philanthropic or social or governmental value. This notion, that open innovation is the first approach and closed innovation the last, is one of those upside down principles.

HASSAN: *Are there any implications of that which might be particularly relevant to the neglected disease space? I’m thinking of all the product development partnerships that have arisen over the last decade or so – there’s lots of virtual R&D happening, and even experiments in open source R&D models. Is there a particular sweet spot you see for neglected diseases where things like InnoCentive could help fill a gap?*

ALPH: I might turn that one around and say, neglected diseases are a sweet spot for open innovation. The reason I would turn that around is because I think neglected diseases have exactly the right kind of architecture of problems and needs that effectively demand open innovation.

The closed innovation model can never be made cost effective enough to chase some of those types of diseases. Yet, at the same time, the scientific challenge for obesity and the scientific challenge for leishmaniasis disease may well be equally stimulating to a researcher. And so you can get intellectual engagement. You can pay in currencies other than cash.

There are open innovation cash reward systems–InnoCentive’s a practicing example of that. But it also compensates in intellectual satisfaction and challenge and social good. I think that someone can bring in all of these different factors and stitch together an ad hoc company–“company” in the sense of the word that it is an integration of different disciplines and capabilities striving to achieve some common outcome. They can stitch this team, this company together on the spot to solve that one particular problem.

HASSAN: *It sounds like we’re feeling our way towards a marriage of the InnoCentive model with open source management methods as they’ve been used in software, and even in neglected diseases as with* [OSDD in India]().

You would have some things done on an open source platform. But when there is an appropriate challenge, you would then invoke InnoCentive as a sort of black box to solve that challenge, and feed the solution back into the open source process. The challenge then becomes, what is the organization or the virtual management team that coordinates all of that to happen?

ALPH: I think you’re describing something that will be a part of our future. With my colleagues like Eric Bonabeau, we mentally incubate this under the code name “hive pharma”. It is a structure like you described.

InnoCentive was built fundamentally as a horizontal play to work in pharma, consumer products, agriculture, food science–across the board, to prove a point around “will people try to solve your

problem for a potential payout?" Will they become bounty hunters–are there a sufficient number of people motivated to take that on in order for the system to work? And the answer is yes.

But there's also a vertical version in which it's essentially a pharma company or an agricultural company or an aviation company using a mechanism like InnoCentive as a way of taking their big challenge, dissecting it into small pieces, sending the pieces through the InnoCentive platform that are appropriate for InnoCentive, sending the pieces through the university platform that are appropriate to university–similarly for contracting platforms, consulting platforms, all kinds of open platforms–and then re-aggregating. A process we describe in the book as "challenge driven innovation."

So I think that, in addition to us saying that companies need to change the way they're working in order to take advantage of open innovation and crowdsourcing, there are other ways these capabilities may evolve. Another path that I can easily imagine, and that I hear you describing, is companies with these concepts built into their DNA may be founded as competitors to older approaches–companies that compete not only on innovation of new products but innovation in a sector's business and research model as these capabilities become easier and easier to access and tap into.

HASSAN: *Absolutely. It's a very intriguing vision for the future. This is really resonating with some of the ideas that, for example, people like* Bernard Munos *have been advocating.*

Alph, turning to our second-last theme, I want to dig into a couple of strategic aspects of InnoCentive. First question on that is, if you look at InnoCentive as a black box, as a platform for health R&D today–what are the key metrics by which you measure its success?

ALPH: Comparing InnoCentive's solution rate with historical values is a little complicated because historically we work on different problems. We do the best we can by grouping them together.

We look at solution rate. We look at novelty. We have conversations with the companies in which we're asking them to guess, "Do you think you'd have come up with this on your own? If so, do you think you'd have it in hand now, or do you think it would be something you might have tried after four other failed attempts?"

So we try to handicap for the total cost of doing something, meaning that we try to bake in the cost of failure as well. And we look at just simple ROIs. What's it worth to you, what should it cost you–we do all of these things trying to demonstrate a value.

I'm quite confident that the proof points are there. But I'm not ready to argue for its ultimate success until sectors of business that provide crucial goods – whether it's pharma or food or healthcare or consumer products – until they have fully embraced open innovation and they have rewired their internal processes to capitalize on what it has to bring them. I consider our real success is still living in the future.

HASSAN: *Aside from basic business economics, what is the biggest challenge which you face with InnoCentive?*

ALPH: I would say that it's selling against the cultural barrier we've been talking about. That's the biggest challenge–the degree to which we cling to familiar ways of doing things, and, to be fair, proven ways of doing things.

I recognize that organizations–whether it's the Gates Foundation, the US Federal Government, or a corporation–cling to the past and proven methods in a healthy way as well. One of the ways that I

describe that behavior is it's homeostasis. It protects you because your environment's going to throw all kinds of wacky new opportunities at you, and if you don't have a little bit of a rejection mechanism and immune response, you're going to get yourself whipsawed into bankruptcy trying to chase after every new thing.

Nevertheless, it does govern the rate at which change can occur. It's governing the rate at which we're moving into a more networked, open organizational structure that I think will reap great benefits.

HASSAN: *Alph, a couple of last questions on the collaborative health R&D sector as a whole. What specific collaborative tools or approaches to health R&D, aside from InnoCentive, do you find promising?*

ALPH: Data banking structures are an important step going forward, in which you have some commonly held databank and you can give widely distributed access to it. Without the ability to share and organize around that data, the efforts of individual disciplines remain too disconnected.

I also think that one set of platforms that is out there but maybe hasn't gotten a lot of attention is the area of serious gaming.

HASSAN: *Serious gaming?*

ALPH: Serious gaming. I don't pretend to be an expert in it, but the mechanisms involved in gaming are about bringing diverse groups of people together to achieve common goals. You can say anything you want about multiplayer online role playing games or video games in general, but if you strip it all away and just look at the underpinnings of what's being accomplished, you can see that there are a lot of collaborative elements being introduced that aren't exploited by the business world.

When I attach the word "serious" to gaming, I mean a usurping of the technologies, the platforms, the sensing systems, a lot of the things that attend gaming. Many of these platforms–and I don't mean platform just in terms of software, I mean platform with a big capital P–could be applied to the world of work.

HASSAN: *Perhaps even* building on early successes like Foldit and so forth*. As you say, games have solved a lot of the motivational challenges of getting people to spend lots of time solving various problems–in fact, getting people to pay you to spend lots of time solving problems. Why not harness it for the common good?*

Following up on that interesting suggestion, do you have a thought as to any catalytic investments that could help collaborative health R&D as a sector?

ALPH: I think that there are some things that fit there. One marker of an opportunity would be the kind of company that you were beginning to get a glimpse of and that I was trying to describe–a completely novel open platform in which you basically manage your R&D process online. And why not do it for neglected disease, because you're not threatening anybody's livelihood? These are generally outside the world of ROI-driven investments.

I said to a large health foundation: "You know, you invest many dollars, but you invest it in doing things the old way. Why not help found a completely different platform for the way drugs are developed? Let's bring out a platform change so that it starts to lead the way, where traditional pharma can then see the proof points and start to adopt a different platform."

HASSAN: *A sort of hive pharma PDP.*

ALPH: Yes. I admit that the real challenge here is finding an amount that would be catalytic, because demonstrating a convincing proof point is an expensive challenge. There's only going to be a handful of organizations or entities that are up to it; maybe a National Institutes of Health initiative.

I do think that if it's proven viable, commercial entities will have to follow because ultimately they're accountable for doing things efficiently as well. But unless there is some demonstrated path, the shareholder is telling them, "You keep your risks within a certain set of boundaries, because I invested in you assuming a certain risk profile."

That's where the role of governments, foundations, and others can provide some of that catalytic money–but I would put it into a structural transformation, rather than yet another disease target in healthcare.

HASSAN: *Lots of ideas to follow up on. Alph, last question for you. Listening to you speak today, and seeing some of the things you've been involved with for many years, you've clearly spent a lot of your life on collaborative R&D issues. What life lesson have you learned about founding successful collaborative platforms–for example, about successfully engaging or organizing a global community?*

ALPH: Well, one thing I've learned in these communities: it's amazing what you learn about motivation when you take the salary card away from a manager. [Laughter]

These are folks that can just as easily go elsewhere. Their brains are very portable. If you want to engage them, you've got to pay them well. Whether that pay comes in the form of feedback, or respect, or intellectual challenge, or whatever form it takes–these are people that deserve to be paid well.

One of the reasons that a network couldn't supplant existing pharma if you had to pay everybody an hourly wage is because, for any given pharma problem we've posted out there, we've got more people engaging from more different perspectives than you could ever afford on a prospective basis. It's the "bounty" property of paying for success that makes this possible.

But those other people are not to be left unpaid. And so you've got to find another currency and pay them in that currency. Unless you want your community to just dissolve and go away, they must be compensated–and that comes in terms of feedback, communication, respect, engagement, acknowledgment, intellectual stimulation–it comes in a lot of forms.

HASSAN: *A point to reflect on. Alph, thank you so much for speaking with us today.*

ALPH: You're very welcome.
