# Supplementary material for: Ten Simple Rules for Cultivating Open Science and Collaborative R&D
Source: PLoS Comput Biol. 2013 Sep 26;9(9):e1003244. doi: 10.1371/journal.pcbi.1003244 (PMC3784487; doi:10.1371/journal.pcbi.1003244)
Supplement: Text S2 — A conversation with Barry Bunin, CEO of Collaborative Drug Discovery. (PDF) [file pcbi.1003244.s002.pdf]

Insider Views of Collaborative R&D for Health: Q&A with Barry Bunin of CDD

April 26, 2012

Barry Bunin is the CEO of Collaborative Drug Discovery *(CDD), a web-based platform for selective sharing of drug discovery data. CDD allows preclinical, biological, and chemical drug discovery data to be securely stored, shared, analyzed, and collaborated upon through a web interface. It can be used to build private, semiprivate, or public virtual drug discovery networks.*

HASSAN: *Barry, thanks so much for speaking with us today. What is the goal of CDD?*

BARRY: Sure. We're focused on open innovation, but from a very pragmatic perspective. Because we are a business, we need to not only have good ideas—they need to be pragmatic. They need to make black-and-white economic sense.

In global health, we have worked with the Bill and Melinda Gates Foundation on tuberculosis. Our work encompasses the full spectrum of working with industry, working with academics, working with foundations, institutes and even government labs.

What we do is we support collaborations. CDD is all about collaborations.

HASSAN: *Can you tell us about your collaborative technology?*

BARRY: What's interesting from a technology perspective is we allow people to have secure data partitioning (selective and fine-grained data sharing) to support different business models and intellectual property rights. The irony is the more that you give people the power of having data secure, and partitioning, and knowing that for commercial interest they can protect it—the more they are actually able and willing to collaborate.

We have the full spectrum: from private for one group only, to collaborative where two or more organizations can work as if one, to completely open in public. The tools have advanced for the chemical and biological data, for the science of discovering new drugs, handling the ADME-PK-tox-animal type data (for pharmacokinetics, toxicity, and so forth). Companies that are purely in the for-profit mode are using this now—like a venture capital funded startup, or an academic lab looking at some commercial area like cancer.

HASSAN: *So what does that imply for drug discovery?*

BARRY: The new part is having it be collaborative in people's natural workflows. You can share even one measurement from one day—one experiment with all the detail of the graph, the image, the numbers, the chemical structures—and do that with anyone around the world.

This has been what's really held up drug discovery relative to progress with the computer industry. There's a lot of reinventing the wheel in our field.

The big breakthrough here was being able to handle the security in data and the IP issues, and giving people the control to make it as private or as collaborative or as public as they want. Control can be temporal control, it can be by project, it can be by data types.

It started with no one using this. A little bit like the fax machine is not very useful when you have one

person with a fax machine, but when everyone has one it becomes taken for granted—ubiquitous. We had 28,000 logins in the past year and have had eight years of a perfect security record hosting the data in the cloud, and so now it's becoming more and more accepted.

Beyond how much or how little people want to share data, there's another challenge which people are generally less aware of: just getting the data into the database. If you can put one compound into the clinic, you will test dozens in animals, hundreds in cells, thousands or millions in enzyme assays. We handle the data on those aspects. The question of sharing data and collaborating openly is one problem that gets all the attention, but the other problem of capturing the data in a useful easy way is just as important.

HASSAN: *It sounds like the controlled collaboration feature is critical for getting people to make use of this. What are the biggest challenges in how to make that controlled collaboration feature work in practice?*

BARRY: The biggest challenge is making it work for people's natural workflows. One might naively think that's simple and obvious, but it's not. You need to do this with accuracy, and with fidelity for capturing the data. You need to make it easy enough that it's not more work to collaborate – so that the benefits exceed the overhead or the activation barrier.

For example, we equip the Rockefeller Screening Center, where they'll be testing molecules—libraries of compounds in microtiter plates, 50,000 of them or so at a shot—with 20 different professors doing groundbreaking research. Each professor needs to be able to see her or his own 1/20th of the data at the screening centers running the experiments. They're seeing the molecules and generating the data around the novel biological insights.

From the screening center perspective, to have efficiency, they don't want to upload 20 times to 20 databases. At CDD, they can upload it once. Like tagging photos in Flickr—you can just put the data into 20 different projects, and each professor sees his or her own 1/20th of the data without even being aware of the existence of the other 19. When people can and should within their natural workflows see 20 projects, they do. When they should only see 1/20th, they do.

There's the collaboration around the data, and then there's the collaboration around the people. With the Internet, you've seen examples where there are different ways to facilitate communication—whether it's Wikis, whether it's Wikipedia, whether it's LinkedIn, Facebook, or even more private mechanisms like SharePoint. I talk about us having fought and won two wars: one is handling all the scientific depth, and two is making the collaborative technologies natural for people's workflow, which I think is the biggest challenge.

HASSAN: *Interesting. Turning to global health in particular, which you mentioned briefly previously, how do you see CDD specifically helping global health R&D?*

BARRY: Well, I see global health therapy as, for us, good existential proof for all drug discoveries—a Trojan horse strategy. In the neglected disease space, you get some of the moral high ground for doing something good for the world, and establish some trust there.

Since we're working on global health so much from day one—in African sleeping sickness and TB with Gates and the EU and the NIH—we have to keep reemphasizing the security and privacy for the commercial areas. But I think people are more willing and interested to work together, and we're actually establishing efficiencies that are going to carry through for all drug discovery.

There are some surprises too. With GlaxoSmithKline—a big pharma—with their Tres Cantos open lab. For their malaria screening, there are 13,000 compounds from their corporate library against half a dozen assays that were shared openly for the first time on CDD. This is a big pharma. And Novartis as well, and we have collaborations with Sanofi Aventis and AstraZeneca with MM4TB.

The pharmas—it's not what you would think, necessarily. Sometimes the pharmas will even collaborate more openly than an academic working on neglected disease or global health. They may have data that they keep private either because they're waiting for their next grant, or publication, or just double-checking their results.

Giving everyone control is relevant for global health as well as commercial areas. I would say always encouraging people to collaborate as much as they can without ever forcing them is the key. I think that's the key for efficiency in global health—and global health means both commercial as well as neglected diseases.

We have the largest set of TB data and probably the largest set of malaria data openly available. And we have other sets like of the known drugs for drug repositioning or repurposing, or from Bryan Roth at University of North Carolina, we have 47,000 measurements against 699 GPCRs (G Protein-coupled receptors) that 40 percent of all drugs hit.

Nature doesn't distinguish between poor people and rich people. Nature's GPCRs might be relevant for infectious disease, for cancer or CNS disorder (central nervous system disorders). So a lot of these things I call therapeutic area agnostic. A collaborative tool for making people collaborate better together—much like nature, it doesn't distinguish. You can have protease inhibitors for cancer, or HIV protease inhibitors for AIDS, and proteases have similar shapes, similar molecules within them.

I think the exciting thing here is using the global health areas to push the envelope—but it's not what people think, it's not just a game for non-commercial areas. There's no reason a priori that an approach isn't as applicable for commercial and humanitarian application.

HASSAN: *Let me push that a bit further, and perhaps ask you to speculate a bit. Do you think that platforms like CDD, by making it easier to share approaches and advances across disease-specific areas which might in the past have been more separate, will help to cross-fertilize approaches more in the future?*

BARRY: I do think it's already happening. The reason is that these are hard problems, and different brains will look at the same information in different ways, because of all their expertise and knowledge and intuition. That's true both of human brains and computer brains, if you will, or algorithms.

The other thing is that our big breakthrough is figuring out that it's about the science and it's about the funding. And so the extent to which we can be a catalyst for other people's science getting better and other people's funding getting better and vice versa sets up a virtuous cycle. This is where CDD has become really exciting from a disruptive perspective. I saw you interviewed Bernard Munos—he is a thought leader for thinking different in this industry.

What we're doing is setting up a virtuous cycle to make that work within the reality of not only today's data and IP requirements but also today's financial requirements. Whether it's industry, whether it's investors, whether it's foundations, whether it's governments—there are ways in which we can work together with folks.

We have this sort of neutral Switzerland aspect to the drug IP. We're not going after the gold, if you

will—we're just developing the tool. We can partner with a lot of groups where they focus on getting the breakthrough discovery, whether for global health tuberculosis or a commercial cancer market. All we want is to make the platform better for everyone. So it's really clean from an IP perspective: we always have 0 percent of the drug IP.

We do want to make the software better, and since that's a win-win, it can really scale now. I mentioned we have 28,000 logins from the past year. You can imagine if this goes up to 100,000, there are going to be lots of examples of synergy on the science side and the business side.

HASSAN: *Speaking of logins, how do you measure CDD's success today?*

BARRY: Via others. Via the amount of collaborations that we catalyze. Our case studies on our website talk about this spinout of Harvard working on HDAC inhibitors, the NIH blueprint working on neuroscience with seven labs, the Gates Foundation working on tuberculosis, the EU with two big pharmas—there are other case studies as well on that website.

We've worked with researchers at Harvard, MIT, Cornell, Columbia, John Hopkins, UCSF, Stanford. Each of these groups is advancing drugs forward, and it's an honor that they trust us and that we provide real value for them. And the number I quote publicly is the 28,000 logins because we track that.

Quantitatively, for example, the spinout of Harvard (which uses CDD to facilitate working with industry and with CROs in China) —they went from a few $100K angel financing level up to something like a $27 million Series B, and filed an IND (Investigational New Drug application) for testing in humans. So that's the story side, the qualitative as well as the quantitative measurements.

HASSAN: *Following up on that, how would you like to measure your success in the future—both in terms of the metrics you mentioned already, and in terms of other metrics which you don't at present measure but which you might want to be able to measure?*

BARRY: We have internal metrics as a company for success that we look at. We have had 140 million datapoints securely hosted on the web, and we have had various labs that I mentioned.

I would say if we had four times the number of users that we have now, and also qualitatively if the platform becomes relevant not just for everyone that we're working with but for everyone else in the industry, even if they're working with other technologies…then we'll become ubiquitous and we'll have had a very broad impact.

Then there will be numerous examples where this matters. You'll start to see an uptick not just in our internal metrics, but in the external metrics for the field—the whole field starting to become more efficient. Then we'll have made a big impact, and had a life and an existence that's meaningful.

HASSAN: *One challenge that I've heard a number of people who develop these collaborative platforms talk about is the difficulty of measuring or valuing large-scale collaborations. In other words, if you have 100 or 1000 people collaborating, then how much value does each incremental bit of collaboration have? Do you have any thoughts on that, or is it even important to measure that?*

BARRY: I think that's the strength of the system that we're developing, with the continuum from the private to the open and everything in between—it allows you to get the best of all worlds. I would say that a lot of the more successful collaborations might be two or three groups working together, and that's something entirely supported with this collaborative approach.

When I started the company, I was going to call it "Open Source Drug Discovery", and that was too radical for the majority of the marketplace. So I turned back to "Collaborative Drug Discovery" so even at a subconscious level you've got the security and privacy of small groups—before patents, before publications, before putting data in PubChem and PubMed and GenBank.

That's my response to it on the one hand. And on the philosophical side, the challenge to collaboration is there's more overhead of herding cats and working together with other folks. The power of it is if you have technology that can make that happen better, and processes that make that work better, then you have this huge benefit—much like a good company where people are working better and it's a healthy organization. It's more efficient.

If you can overcome the challenge of collaborating, of working with others, you do get a significant dividend and a significant reward on your effort. You also get the unexpected. You get the things that you never had happen before. I agree it may be harder to track, but for solving the problems in nature faster and economics for drug discovery faster, it's not just a little thing—it's the key thing. We have to figure it out in the industry.

HASSAN: *Right. Looking ahead, aside from funding, what do you see as the biggest challenge facing CDD?*

BARRY: Scaling. This is true of any company. When you start out you get really brilliant hardworking smart folks doing things, and as you grow, you have to maintain that quality.

On our About Us page, we have four areas where we're going to need new individuals for writing grants, for developing the software. When we're developing the software, we're competing with the Googles and the Facebooks of the world. Also helping people on the data and science side—in all of those areas, we need to maintain quality and we need to grow.

Those are the challenges moving forward—to figure out, "okay, who's the very best next talent?" Companies go through these phases where things can get harder and easier, and it has to do with complexity and challenge of managing lots of folks.

I think the fact that we're externally setting up things to collaborate well means that internally we are as well. We use GoToMeeting, we use Salesforce—we use all sorts of other technologies that leverage the biggest change of our lifetime, which is the Internet.

HASSAN: *Let me ask an offbeat question: when should CDD not be used? For which situations is it not appropriate?*

BARRY: Well, I would say there are domains that we don't handle. For example, we focus more on the scientific data and more on the pre-clinical drug discovery side of things.

The other aspect is the economic question of cost-benefit for folks. I think we're very good at being easy to use. There are some more sophisticated specialized tools, but we're more differentiated on the ease of use and the collaborative aspect in the cloud.

There are going to be cases where people have traditional systems internally, and they need to judge for themselves the cost-benefit of using CDD versus maintaining the status quo. I think that bar is moving in our favor over time, but it's best for everyone to judge for themselves.

HASSAN: *All right, last question: aside from CDD, which other collaborative tools or approaches for collaborative health R&D do you find promising?*

BARRY: One of our technology partners already in CDD is ChemAxon in Budapest, Hungary, and so we have all their tools within CDD. We have ADMEdata.com to try and predict drug development properties—that's useful if folks want to look at the drug development side of things.

Other technologies? GoToMeeting we use everyday five or six times, where we can securely share our desktops. That works really well in complementing CDD because we can share our desktops, and show the power of technology within our niche of drug discovery with some general collaboration tools. Internally we use Redmine as the wiki and that's open source software. Those are just a few—I could list dozens of others as well.

HASSAN: *Thank you so much, Barry, for taking time for this!*

BARRY: You're welcome. I think it's great what you are doing.
