# Supplementary material for: Ten Simple Rules for Cultivating Open Science and Collaborative R&D
Source: PLoS Comput Biol. 2013 Sep 26;9(9):e1003244. doi: 10.1371/journal.pcbi.1003244 (PMC3784487; doi:10.1371/journal.pcbi.1003244)
Supplement: Text S3 — A conversation with Leslie Chan, open access pioneer and director of Bioline International. (PDF) [file pcbi.1003244.s003.pdf]

Insider Views of Collaborative R&D for Health: Q&A with Leslie Chan

October 4, 2012

Leslie Chan is the Director of the Bioline family of open access bioscience journals*. He is also a scholar at the University of Toronto who has* written widely about open access.

HASSAN: *Leslie, tell us the story of Bioline International.*

LESLIE: I'm the project director right now. Bioline was founded in 1993, in the very early days of the Web, by microbiologists: Barbara Kirsop from the UK, and her colleague Vanderlei Canhos from Brazil. Because many of their colleagues were from developing countries, they were quite frustrated with the fact that they couldn't read each other's publications.

So they thought, "Oh, this new Internet thing should be useful for sharing data, information, and published articles". They began Bioline, and it was Vanderlei's Brazilian organization CRIA, or Reference Center on Environmental Information, that provided the initial server and continues to do so. From the beginning, it was truly a collaboration between the global South (lower-income countries) and the global North (higher-income countries).

HASSAN: *It sounds like back in 1993, Bioline was really a pioneering initiative–long before better known open-access initiatives like PLOS Medicine. What's the big problem that you were trying to solve with Bioline?*

LESLIE: The big problem was that a lot of these journals from developing countries were simply invisible. Number one, they were not indexed by the mainstream indexing databases, so they were invisible to international researchers. Number two, in those early days, many of the journals, which wanted to provide access, didn't have the technical know-how.

So in the early days, Bioline was really a technical partner to ensure that those without the technology or the bandwidth – which many still don't have – could make their content accessible over the Internet.

HASSAN: *Why is that important for health R&D?*

LESLIE: When we're talking about health R&D, we're talking about health issues that are global in nature. When you're looking at SARS or other emerging infectious diseases, they're not really localized anymore.

In global health research, I believe that until recently most researchers were working with an incomplete understanding of these diseases, because their data were limited to accessible publications–mostly from the North.

We have very valuable data in journals that are published locally in developing countries. These data are not being used, understood, and integrated into overall pictures of global health. That has been a major problem.

HASSAN: *What I'm hearing is that it's important to ensure that local knowledge, observations, and science is known and used by researchers worldwide.*

LESLIE: Right. That was one of our initial goals: to make sure that knowledge from the South is being

integrated into a global system.

But then as we went deeper into the project, we realized that for a lot of these health issues, it is also important that developing country researchers can access other research from developing countries. It's no good just having access to journals from the North. Researchers need to have access to research from other parts of the developing world with similar socioeconomic and ecological conditions, since this is more relevant for their understanding.

HASSAN: *Not just South-North information flow, but also South to South collaboration.*

LESLIE: Right–even within the same country. I remember examples of researchers in India who were down the road from each other, yet who couldn't read each other's research until it became open access.

HASSAN: *Do you have any specific stories which illustrate the value of open access for R&D?*

LESLIE: There's a journal that is based in Uganda called African Health Sciences. What motivated the editor, Dr. James Tumwine, of that journal to start the journal was that many diseases like sleeping sickness specific to that part of the world were not really talked about or published in international journals. And even if there was literature, it was often too expensive to access because it was in expensive subscription journals.

So part of the reason Dr, Tumwine started *African Health Sciences* was to provided a venue for research of specific relevance to that area, like sleeping sickness or more recently the nodding disease or epilepsies. This research often cannot get into international journals because of its limited audience.

African Health Sciences started in 2006, and has been publishing plenty of papers on local diseases and condition. They even had special issues on post-traumatic stress disorder for child soldiers, for example–hard to find anywhere else. I did a very quick search recently about sleeping sickness, and found many key articles that were citing articles published in *African Health Sciences* as a foundation. That is an important metric establishing that there are researchers in that region doing important work, and that they are being built upon.

HASSAN: *What would you say would be other good metrics for looking at the impact of Bioline?*

LESLIE: This is the million-dollar question, right? Some are fairly standard: usage and the geographic distribution of users. Last year, for example, we had over 5 million full-text downloads throughout the system. That tells us that a lot of people are reading the articles. The readers are coming from all parts of the world, and we have a lot of access from key universities, including many from the Global South.

Now what we would like to know is, what are people doing with these articles after they download them? Are they building on them in their research? We can do that through citation tracking.

But more importantly, we're not interested just in citation–we're interested in real usage. Are the articles being used for training new doctors or new researchers? Are they being translated into health promotional or teaching material? Are they being used in policy discussion and to inform debates?

HASSAN: *That seems like a key distinction. The metrics you mentioned before were more about quantity of usage, whereas these latter ones are about quality of usage.*

LESLIE: Yes. This is about what we like to call engagement and influence.

Increasingly, as we know in the social Web, things get linked and repurposed in unexpected ways. We

would like to track some of this flow of information and see how people use the material.

HASSAN: *How do you actually measure that?*

LESLIE: Well, right now we don't have a good way to do that. I know a lot of journals are beginning to track Twitter feeds, blog mentions, Facebook likes, and all that stuff. Many journals are using these kinds of social media metrics. They are built into each article as a way to track this kind of article-level metrics.

HASSAN: *Although that's still simply tracking people talking about the article, versus how much they actually use it for doing some research or other activity.*

LESLIE: Right. At a recent talk when I was at the University of Cape Town in South Africa, my colleague Cameron Neylon who works for Public Library of Science showed an interesting example of a paper on HIV/AIDS. It was published by a researcher from the University of Cape Town, and through Twitter Cameron was able to track mentions of that paper by prominent NGOs based in South Africa, and these NGOs then further spread that article to other NGOs.

Cameron was interested in asking questions like, what did these NGOs use the article to do? You can email these people and say, "What are you doing with that article? What was that article used for in terms of your own organizations and staff?" You can follow through with this kind of contact.

HASSAN: *A sort of snowball sampling, both over groups and over time.*

LESLIE: Right. And in fact you can track the network of how this gets spread.

HASSAN: *If we think specifically about the example of developing a new health solution, I'm wondering if this suggests ways to look at the chain of research which might have been used during the R&D?*

LESLIE: Right. That would be the kind of network analysis that one might do in the future. It's beyond simple citation. It's a network approach to the influence of a particular piece of research.

HASSAN: *Let me ask a related question. There has been a lot of discussion about the value of doing more data sharing. When we don't share data, we have lots of duplication of various sorts–all the way from basic research to clinical trials. So, in terms of open access for data, what advantage do you think Bioline or open access more generally might offer for making data more available?*

LESLIE: This is critical for research in developing countries. As you might know, a great deal of research never gets to the publication state. Many of my colleagues tell me that in sub-Saharan Africa as much as 85 to 90 percent of research conducted never sees the light of day.

What happens is that you have a lot of data that has been gathered for one reason or another, and they simply sit on computers, or even on papers somewhere in the back room. For years, I've been thinking that this is a treasure trove. A lot of these data need to be captured and then standardized so that they can be shared.

A simple example would be clinical observation of HIV/AIDS. There are many medical researchers I spoke to who are really very busy, with their practice and teaching and so forth. They don't have time to publish, but they have recorded a lot of these observations. What if these observations were collated and made available in an online resource someone founded? We would see consequences emerging that we had never even considered.

HASSAN: *Absolutely. I guess one way that may happen in the not too distant future will be technologically-enabled observations from devices and so forth, where a diagnostic device might automatically record and share those kinds of anonymized observations.*

How might we encourage or support people to share those kinds of personalized observations on things like HIV/AIDS?

LESLIE: One way to encourage this is to build it into the funding framework. For people to do this, you'll need to provide the funding to do it, and the tools to do it. Right now, both are lacking.

I think funding agencies need to rethink how they fund research. Rather than just requiring publication of the research output, data gathering and sharing should be integral to the entire process.

HASSAN: *So you're suggesting that just as some funders build in a bit of funding for open access journal publication fees, they would also have a bit of funding set aside for data-sharing support–and also provide an easy-to-use set of tools to make data capture and sharing easy. Do you think that would result in a much larger pool of observations, which researchers might then be able to use, combine, collate, analyze, and so forth?*

LESLIE: Absolutely. Because once these data are available, then they can open up to different expertise. This is where it becomes very exciting, right? If you're collecting blood samples for malaria diagnosis, then maybe the molecular biologist can look at it, maybe the epidemiologist can look at it, and maybe even social scientists can look at it and then come up with different interpretations of the results.

Right now, publications are not open to interpretations other than what the researchers suggest most of the time. So by having data open up, I think you'll open up to all kinds of new tools and interpretations, which is really exciting.

HASSAN: *That's right. Leslie, if you set aside the very important funding issue, what's the biggest challenge which you face with Bioline?*

LESLIE: One of the problems we've been facing is unfortunately chicken and egg: because of lack of resources, we haven't really developed Bioline into a more modern system. There has been a lot of technological change since we started it in 1993. But if you look at our system, it's still very much a web 1.0 platform.

For years, we wanted to turn it into more of a web 2.0 platform with different services. We talked about article level metrics, citation tools, visualization tools, data-sharing tools. What we really would like to do is bring the system up to date, and allow our partners to enjoy the kind of networking potential that we talked about.

HASSAN: *It sounds like, although we have lots of open-access journals, one challenge is developing the journal systems themselves behind the scenes.*

LESLIE: Yes. Even in terms of licensing, we have a tough time making sure that people understand reuse rights and so forth.

At the same time, the article is an artifact of a few centuries ago–and things are changing rapidly. In a few years, we probably won't know what a journal is. We will have different platforms that allow more rapid and integrated scientific communication.

HASSAN: *Speaking of platforms, let's say that right now I was in the developing world and wanted to*

start an open-access journal. Suppose also that instead of developing a whole new journal system, I wanted to use a modern and up-to-date system that already exists. What would the best possibility be–one that is well-supported, and will evolve into the future?

LESLIE: The question seems to presuppose that we want to replicate the existing journal system. Do we want to advise our colleagues in the developing world to replicate a journal system that we think is on the way out? Or do we want to encourage them to adopt something that is far more current–that is cutting edge and is going to lead the way?

Now, the problem with the latter is that they're apprehensive about being too far-out, because the global evaluation system is still very traditional. So I'm constantly being asked, "What's the impact factor of the journals on Bioline?" This reflects the conservative mindset that is still driving the current system.

HASSAN: *It sounds like the metrics which large international funders and donors are using to evaluate the open-access platforms themselves are shaping the kinds of tools which people are using–or not using.*

LESLIE: Exactly. I often argue with funders that they need to think broadly about the different kinds of metrics that are enabled by networking tools.

Suppose they were to tell fundees, "Okay, we will now value different kinds of engagement and influence. If you adopt a system that allows you to communicate your research and share your data in ways that can show engagement and influence, then we will reward and recognize your research."

Then people will be more prone to try something different from the existing system. I think it comes to realigning the value system of what get rewarded.

HASSAN: *Absolutely. Let's return to the question of, "What would you use today?" What would you recommend that somebody in India or Africa use today as an open-access/open-publication platform, in the more traditional way?*

LESLIE: There's a very well-recognized open-source journal system called OJS, the Open Journal System. And Open Journal System allows you to quickly install the system and manage the workflow of peer review, and then publish traditional journals.

A number of organizations have started doing that. They have been publishing their journals or migrating their existing print journals into OJS, and making them available online for open access.

That system has a worldwide community of users. It has been localized into Portuguese, Spanish, and French, for example.

HASSAN: *Let's take a minute now to look at what you've done with* OASIS*–your other initiative which is like an overview of open access. Could you tell us briefly what it is and what impact it has had?*

LESLIE: OASIS started because my colleague Alma Swan and I felt that there's still a lot of misunderstanding about what open access is. So our thought was, "Okay, we'll outline the key issues for someone who may not be fully aware of these issues, to get a good primer so that they can probe for further questions."

We also at the same time wanted to provide case studies. If they want to go open access, what are the business cases? What are the options available to them? They don't have to feel they're alone in doing

this. There are a lot of people doing it, and there are many lessons learned already–and we can share those lessons.

HASSAN: *And based on having done it, what advice would you have for others who are making primers?*

LESLIE: Well, one of the things about a primer is that it can easily become outdated. We were quite happy with this two or three years ago, but when we look back now, a lot of it needs to be updated.

And so the key lesson there is, have some system built in where there are resources devoted to updating and maintenance, and to linking up to emerging initiatives like yours that have crossover objectives.

HASSAN: *Good advice. Leslie, let's turn in our last few questions to looking at open access and global health R&D more generally. First of all, could you summarize how you feel open access can best help global health R&D?*

LESLIE: One of the things that I mentioned earlier is access to the literature. Number two, to me, is participation of the researchers themselves.

If your research is not being read, you're not participating in a global dialogue about the research areas that you're engaged in. This isolation has proven to be very challenging and detrimental to research in global health, particularly in developing countries.

When you allow researchers to be read, interesting things happen. They're being consulted. They're being invited to join research networks and conferences. These are tremendously important in terms of building local capacity and international collaborations on issues that affect people across the globe. So participation really is a key open access advantage.

HASSAN: *One could imagine that to ensure that a new vaccine or other result of health R&D gets taken up, there could be cultural, economic, and ethical issues that occur downstream. Those could short-circuit the whole effort, as we have seen in examples like polio eradication.*

And so do you think that enabling more participation from developing country researchers could lead to a better "early warning system", which ensures that expensive health R&D efforts can mitigate downstream risks?

LESLIE: Absolutely. A lot of really expensive studies are done by researchers who are flown in from the North to a certain region, and then they go back and do the analysis and publish the result. The system of research and publication is such that problems may not be spotted until two years later. By then, they're being made into interventions and policies.

If those processes were made open early on, a lot of local health workers, researchers, or even people knowledgeable with the conditions could easily spot problems.

HASSAN: *A sort of radar, if you will. Leslie, what specific collaborative approaches aside from open access do you find promising?*

LESLIE: Many researchers from the developing world are on social networks of various kinds. They're able to meet researchers and offer their insights and, by the same token, learn from other researchers from other parts of the world. To me, that has been transformative.

What's interesting too is that the collaboration often now goes beyond disciplinary boundaries. For example, text mining is being brought to all kinds of research data that are generating interesting

research questions that would not have been asked before. Text mining started out with text, and then went into genomics, and then genomics created new tools that allow us to go back to interrogations of other disciplines. I think these are really promising.

HASSAN: *Looking forward, do you see any catalytic investments which could help collaborative health R&D as a whole?*

LESLIE: I keep going back to the reward and incentive system. It's not even putting in new money as much as realigning the value of what's recognized.

Our R&D system now doesn't much encourage collaboration. It more encourages competition, because it's often a winner-take-all approach to funding–so you want to keep your result and your approach away from your competitor because you have to win the funding. The citation system also doesn't encourage collaboration because citation is based on individuals.

HASSAN: *So what should we do?*

LESLIE: Well, I think we need to somehow come up with better metrics that reward collaboration and engagement as part of the system.

HASSAN: *It sounds like you're recommending that funders and donors should use metrics that reward research efforts based on questions like, how many people are engaging with the research process and outputs? How much is the research used downstream? Those kinds of metrics would then change professional and monetary rewards, and reshape the incentives of those doing R&D.*

LESLIE: Right. I think that development agencies are recognizing this. For example DFID in the UK, IDRC in Canada and The World Bank are increasingly engaged in the "open development" agenda that includes realignment of incentives to encourage innovative R&D.

HASSAN: *Leslie, last question for you. What life lesson have you learned from being involved in Bioline, OASIS, et cetera, about making a global scale collaboration work?*

LESLIE: Well, the challenge really is that people are very driven by incentives, and unfortunately these incentives are still bound by institutions and traditions. So it's very hard to encourage people to truly collaborate, because in the end, they need to watch out for their own funding and careers and be able to sustain their own research agenda.

If the system doesn't allow them to move beyond the traditional framework of competition, it's really hard to motivate people to collaborate. That's still a key challenge.

HASSAN: *The importance of getting the incentives right. Leslie, thank you so much for speaking with us today.*

LESLIE: Thank you, Hassan.
