# Supplementary material for: Ten Simple Rules for Cultivating Open Science and Collaborative R&D
Source: PLoS Comput Biol. 2013 Sep 26;9(9):e1003244. doi: 10.1371/journal.pcbi.1003244 (PMC3784487; doi:10.1371/journal.pcbi.1003244)
Supplement: Text S4 — A conversation with Aled Edwards, director of the Structural Genomics Consortium. (PDF) [file pcbi.1003244.s004.pdf]

Insider Views of Collaborative R&D for Health: Q&A with Aled Edwards

November 1, 2012

Aled Edwards is the Director of the Structural Genomics Consortium, which has deposited more than 1400 high-resolution structures of medically relevant human and parasite proteins into the public domain—a significant portion of recent global output. Aled is also an advocate for Arch2POCM, a new virtual organization with buy-in from pharma and researchers that aims to openly develop novel compounds through Phase II clinical trials.

HASSAN: *Aled, could you describe for us your relationship with Arch2POCM and SGC?*

ALED: With SGC, which was launched in 2003, I am the founding CEO. I've run it for the last nine years in its evolution from an organization that focused on generating protein structures for the public domain to generating a larger and more varied set of reagents into the public domain.

The Arch2POCM was a concept that a few of us wrote up in 2009. It got a lot of momentum when Steven Friend joined the team and we changed its name to Arch2POCM and had some meetings among pharma execs and academics. We will soon launch a few programs under that flag—to take a molecule all the way from an idea to a proof-of-concept clinical trial without ever filing for a patent.

HASSAN: *What's the big problem that you're trying to solve with Arch2POCM?*

ALED: I think that the problem, which is a joint problem between industry and society, is that the drug discovery R&D enterprise—that includes academics and for-profit organizations and governments—has not been producing new medicines for many of the more terrible diseases. And if you unpack what the reasons are for that, there are several.

One is we have a very poor understanding of human biology, and thus when we go into clinical trials to test our novel hypotheses in humans, nine out of ten fail. Now, that wouldn't be bad if the failures were completely transparent, but they're not. As a result of the profits we put in place to reward innovation, companies rightly feel the need to have to protect their intellectual property and not disclose any trade secrets in the process.

As a result, what happens is that many companies pursue each idea—and remember, the probability of success is less than ten percent, and none of the information is shared. So we have a process in which ideas fail, resources are duplicated, and there's no learnings from it.

To make matters worse, each of the companies tends to focus on a narrow spectrum of ideas that are generated by the academics. The academic system, in turn, is at fault for focusing many resources on a small fraction of the human proteins. There tends to be a real crowding of activity around certain "hot targets"—and then industry goes to the meetings, listens to the academics, reads the literature, and then in turn focuses on those same "hot targets."

So the total system—academia, government labs, industry—is focusing a lot of resources in duplicate, in secret, on a small fraction of the available human potential targets. As a result what you're seeing is industry shying away from areas like neuroscience that are terrible burdens on society because they fail too often and it's too expensive.

There's not a simple solution because the players are very entrenched and the systems have been set up to preserve the status quo. And it's not that it's an evil status quo—it makes perfect sense, except it's not working. (laughter)

HASSAN: *So what do you think might work better?*

ALED: We need to develop a new systemic way of bringing new ideas to the fore. Why don't we increase the number of opportunities for drug discovery by testing them not in triplicate et cetera, but once or twice transparently and in the open?

Nine out of ten of these novel ideas will fail, but at least we know they failed, and everyone will see why. And the one that's a success will provide opportunities for industry to compete. That's the general concept.

The place at which we intend to stop is at what they call proof of clinical mechanism (POCM), where we have a good idea that if we inhibit that protein or that mechanism, it will have a positive therapeutic effect. That's an important go/no-go in drug discovery. You want to know what works in human beings—not what works in a test tube, not what works in a mouse. That's where "R" stops and "D" begins in R&D.

HASSAN: *Lots to dig into there. It seems like in terms of the challenge, there are really two fundamental pieces to it. One is duplication, and the second is over-focus on a few hot targets.*

To dig into that duplication challenge a bit more, is duplication happening because companies have no incentive to help other companies—or indeed, would rather that other companies spend money on useless avenues and therefore become less able to compete?

ALED: There's definitely a little bit of that, and it's a little bit of making sure the competition doesn't get the market first. Remember, we reward first to market.

HASSAN: *Right.*

ALED: So, any hint of letting the competition know something you know is bad for business, in the current way we set up the reward system. You cannot blame companies for not wanting to share their data because unless everyone shares it at the same time, they are going to lose.

And so far as focusing on the same set of targets—again, you can't blame them. The pharmaceutical industry is science-based. It makes perfect sense to invest your resources where the science is most known, and so the hot targets are the ones for which there is a robust and large set of data.

If professors publish on the same stuff over and over and over again, industry is "forced" to work in that area because it's a complete risk to work on something for which there's no literature. And as a result, all the companies get the same literature, they all get the same journals, they all have the same decision trees—so they all focus on the same stuff.

HASSAN: *How would Arch2POCM help with this "hot target" challenge?*

ALED: In Arch2POCM, one would explicitly focus on completely novel and highly risky targets that no company in their right mind would do internally.

Arch2POCM would start at the very beginning in a completely open and transparent way, knowing that it's going to fail most of the time—but at least if it fails companies and society can tick off that target, cross it off the list of potential ideas and say, "You know what? That doesn't work."

HASSAN: *And in terms of the actual process you use to decide that a target is worth your focus, is that just a matter of how much it's been investigated previously?*

ALED: Yes—not how many patents, but how much it's been investigated. Are there any genetic links to the disease that might give you some early hints that it might be interesting? Things like that. Stuff that eventually every company would use in its decision tree, but at a much earlier stage than any company or even academic or VC would invest resources.

HASSAN: *Right, and because Arch2POCM is looking for those non-hot targets, presumably that also helps you in a social sense because you're not treading on other people's turf.*

ALED: That's exactly right. We call them "pioneer targets" because they're really forging completely new areas. And the idea is that if we can eliminate nine out of ten of them, that saves every company who would have tried it 30 to 50 million dollars.

It increases the amount of knowledge of human biology in the public domain, because all the molecules and data go out there. And for the one in ten that's now clinically validated, it gives companies the opportunity to compete with far less risk.

HASSAN: *In terms of the investigation of a particular target, let's just walk through that briefly. If you start investigating a promising target, how much time and money does it take to get to that POCM stage?*

ALED: Maybe between 15 and 25 million dollars. That's over three to seven years, depending if you're lucky or not.

HASSAN: *And typically would this be done by a single lab, or would you have lots of collaboration happening?*

ALED: Oh, there would necessarily be a network of labs working together. That's another powerful reason to do it in the absence of ownership or intellectual property, because as soon as you have to engage in multi-institution or multi-country collaborations, you're into the area of needing legal agreements to cover it, if there is a reward at the end.

If the agreement is simply to generate scientific knowledge, there are already pre-established ways of communicating. Professors collaborate across countries all the time. The key is to ensure that there are no pacts necessary, which makes it really easy to collaborate—it's just about discovery.

HASSAN: *So after that three- to seven-year period, 90 percent of the time you have a failure in the sense of drug readiness, and you stop.*

ALED: Yes. Now, to be fair, it's usually not black and white. Usually it's "we didn't hit our end point in this disease." Maybe that target would be important in another disease, but at least for this one, it's out. But the compound that we will have made will be available to anybody to test in any disease, and that's another advantage of doing it in the open.

The potential availability of the clinical drug is worth highlighting. When companies have intellectual property around a potential drug, they're quite careful about who gets access. This is rational. If a third party does a poor experiment and concludes, "You know what? This drug causes hypertension,", the company will likely be forced to do additional experiments to reverse the damage done by the crappy experiment. This could cost tens of millions of dollars or more.

And so companies—for very good reason—keep very close strings on their compounds in

development. What Arch2POCM says is, “We'd be happy to let the world decide whether it was a good experiment or not,” if the guy found hypertension. We will, to the best of our ability, make the compound available to anybody to run a safe clinical trial, provided they have a good idea to get it by their ethics board and provided they can raise their own funding for it.

As a result, you get this crowdsourcing of ideas made possible though the release of compounds that customarily would have been patented and kept in house.

HASSAN: *How does the transition work which you mentioned previously, where in the one in ten cases where it looks really promising, there is this transition to a more commercial space? If you're doing all this in the open, then what does the company get?*

ALED: The harder question is, what does one company get? The easier question is, what do companies get?

Companies get an idea that they know is de-risked, in which they can now invest internal resources on making a medicine. In practice, that’s how the world works today. When a target is validated, that spawns dozens of competitive efforts. The first Statin to make it to market was developed by Merck in the 1980’s. Do you think just because Merck was first to market, all the other companies went, “Oh crap, we’re not going to work on Statins. Merck got there first.”? Absolutely not. The “validation” of the target of the drug by Endo in Japan and Merck was the key to new innovation and profit. Dozens of companies are making billions of dollars, and Statins continue to be approved today.

All companies really want to know is, tell me a target that works in a person. Then they can decide whether to compete. They may set out to to make the best medicine, or make a safer one than the competitor. Perhaps they may market a once a day ingestion, rather than the competitor’s three times a day dosing. Perhaps they may choose to innovate in other ways; for example by combining the freely available drug with their proprietary drug to make an even more efficacious combination. There are so many ways to make money on de-risked targets.

HASSAN: *Opening a whole space, really.*

ALED: Yes, absolutely! It's exactly what will happen. We will create markets that others can compete for.

Now, the more unlikely case is the specific molecule that Arch2POCM derisks happens to have every single characteristic of a great drug. It's not patented, but there are many avenues to profit. The potential scenarios are rather complex and in-depth, but I will walk you through them.

Our key to developing a freely-available compound is to exploit the existing laws of data exclusivity. As you know, the clinical trial process is regulated by government agencies, such as the Food & Drug Administration (FDA) in the United States. The purpose is patient safety, and a whole legislative and bureaucratic—and bureaucratic in the good sense—environment has grown around clinical trials such that governments can protect their citizens’ safety.

To test any molecule in a person, you have to abide by these formal steps including creating a database called an Investigational New Drug (IND) application, which documents everything you’d want to know about that molecule, all the tests you did, et cetera.

HASSAN: *A huge dossier.*

ALED: Even though an Arch2POCM molecule would not be patented, no one has the right to run a

clinical trial with that molecule without having the IND database. So in essence although the molecule can be given away and used by everybody, the owner of the IND database “controls” its use.

So what we plan is to have Arch2POCM warrant to disclose all relevant scientific and clinical data, but to “hold back” some of the more bureaucratic fields, the ones that have very low probability of having any impact on scientific understanding. By controlling the database, Arch2POCM will de facto have exclusivity on the right to do human trials with that molecule. Although the molecule and the resulting science are in the public domain, the right to take it into clinical trials is not—or rather, is at our discretion.

Now let’s pretend the molecule looks great, and we say, “Uh-oh. This looks promising. Now somebody's going to have to pay 200 million dollars to test it in a larger patient population, to do the larger clinical trials that are part of the regulatory process of drug discovery.” In this case, we can (if we want) give exclusive rights to access the database to one organization. If the molecule is successful and is approved for marketing, this provides the organization who funded the clinical trials with years of market exclusivity.

HASSAN: *Oh, I see where you’re going.*

ALED: It is this data exclusivity provision that is the key to developing a molecule that is not patented.

The regulators grant rights to market a drug based on the data in the IND database. Existing laws state that those data belong to the organization that generated them for 5 years in the United States, 10 years in Europe, etc. This is a powerful way to gain market exclusivity, even in the absence of a patent. No competitor can market the drug without conducting their own trials, and this is prohibitively expensive. Therefore, the organization that was granted ownership by Arch2POCM will be able to recoup their investment in the Phase III trials. Indeed, in this period of exclusivity, a good drug could make over 2 billion dollars, patent or no patent. This is a good return for a de-risked program.

Generic manufacturers will eventually enter the equation, but they enter only when the regulatory data become public, and they can refer to the other data rather than having to incur the costs of a trial themselves.

And so the idea is that for the Arch2POCM compounds that work, we would give or sell somebody the right to take that compound into phase two and phase three trials. If they are successful—there's about a fifty percent chance—then they would benefit from existing laws and have exclusive rights to sell for five, eight, or ten years based on the exclusivity provisions of the jurisdiction. And if you do the mathematics, it's highly profitable.

HASSAN: *Interesting. It’s a slightly complex process, but it seems to hang together and match the regulatory and legal system that we have.*

ALED: Takes advantage of it, actually.

There is a plausible and pragmatic way of doing it, and all the players are in complete alignment with this. I think it's going to be a very powerful way of opening up new areas of human biology.

HASSAN: *Speaking of all players being in complete alignment, what are some indicators of Arch2POCM's traction so far?*

ALED: The concept has got excitement from at least five big pharmas. It's got excitement from a number of funders.

It's a matter now of circling the square and getting specific projects up and running.

HASSAN: *Great! Of course, money's always an issue—but aside from attracting funds, what do you see as the biggest challenge you face with Arch2POCM?*

ALED: Attracting funds. (laughter)

There are so many norms that are being broken by this. In many organizations, how it works is you've got a few naysayers. That slows down stuff. And everyone can find fault in it not necessarily because the idea is wrong, but because it breaks their vision of the world. So it all does come down to funding, and the recession hitting at this time was probably not the best time either.

HASSAN: *In terms of the difficulty, it sounded like the challenge was getting people to buy in because people are skeptical.*

ALED: The way society allocates funds is rather traditional, and so the problem is finding the public funders that have the chutzpah to do it. The companies will participate because they leverage their funding; it's a relatively small investment for any one company.

It moves the traditional barrier of competitive and pre-competitive, as a pilot that explores if this really works. And then it's up to us to make sure that the sharing mechanisms work, and we're going to learn as we go where the choke points are in the concept.

HASSAN: *Right. If you look forward three to five years, how do you anticipate measuring or tracking Arch2POCM's success for health R&D?*

ALED: That's really interesting, and I have thought a lot about it. There are many fronts on which you can measure success. The simplest one, in terms of ROI for the public funds, is how many publications come up, because that's the traditional metric of NIH grants, of CIHR grants—what's the quality and impact of the publications. Because Arch2POCM will be all about sharing data, publications should be the front and center metric.

The other one, I would say, is speed and cost to a clinical proof of mechanism. The idea is that if you do it in the open by sharing resources, by minimizing legal agreements, you should get there faster and cheaper on average than an internal program. Now, of course, the problem is that it may not be managed as well, because if you're a single organization you can turn screws, right? So there is a potential that it could even be slower, but we'll find out.

Another longer-term metric is whether you indeed effectively kill or validate a mechanism that causes a market to open. The other metric I think we could use would be the relative involvement of patients.

HASSAN: *Of patients?*

ALED: Absolutely.

One of the key problems in drug discovery is the time it takes to fill a clinical trial. That's why it costs a lot—if you could have 1,000 patients today, it would cost a lot less than having to recruit them over three years and pay all those study administrators.

Imagine what would happen if we said to cancer sufferers, "Our potential breast cancer drug is being studied in the spirit of transparency and openness." I suspect that we would fill the trials rapidly, which translates to an enormous saving of time and money.

Moreover, if one gets the patient groups as active participants in the process we should also get more

funding, as there's charities that will get behind the effort.

And so I think that patient involvement as measured by funding, as measured by speed with which the trials are filled—I think those could be interesting metrics. It's certainly something that one should aim for. The attraction to patients is the knowledge that Arch2POCM's mission combines novel drug discovery with complete disclosure of the information in real time. The effort truly serves the public good.

HASSAN: *Absolutely. It's like a high-commitment version of open access for pharmas. And in fact, it seems related to John Wilbanks's* Consent to Research idea *as well.*

ALED: That's it.

HASSAN: *What do you see as Arch2POCM's potential relevance to global health R&D in particular?*

ALED: Arch2POCM will know no borders. Science funding currently is very parochial; few funders fund outside the borders of their own country. Companies on the other hand don't know borders. So for Arch2POCM, the particular advantage of getting industry funding is that you can spend it anywhere.

And so for global R&D, the Arch2POCM concept, with the involvement of industry and potentially patients, I think will allow us to do the science anywhere it makes sense—and that includes the developing world. That will be a tremendous advantage, and I'd be really excited about that.

HASSAN: *That's an interesting perspective. Since Arch2POCM has a collaborative approach baked into its DNA, I'm wondering if that gives it a particular niche or advantage for low-market diseases?*

ALED: It is equally applicable to low market diseases—not especially more or less applicable.

People argue that industry should invent medicines for neglected diseases because drugs are invented in the commercial sector. That's unfair to the companies. Investing in medicines for diseases more relevant to developing countries should be a shared effort. The lack of medicines is a global health problem that's causing economic burden on the developing world, so why shouldn't the World Bank or the IMF contribute? Why shouldn't Canada help fund it? If by sharing we can make more children healthy and spend more time in school, those countries will increase their standard of living, and we'll get a healthier world with fewer inequities.

HASSAN: *So think of it as more of an international development issue.*

ALED: Yes. It is important to involve industry because they know how to do it, and it's best done in a regulatory environment that's more controlled. But putting the financial onus on industry to develop these medicines is slightly misguided, I think.

It may make little sense for a big pharma to take the risk to develop a trypanosome drug. Governments should participate more. I think we, as citizens, are shirking our global responsibilities by pointing the finger at industry.

HASSAN: *Intriguing perspective. Aled, I want to make sure we have a chance to also bring in SGC (the Structural Genomics Consortium) briefly. You led the SGC from its inception. I'm curious if you see any lessons from SGC for Arch2POCM.*

ALED: In my opinion Arch2POCM is a natural evolution of the SGC. So it's not lessons; SGC provided the model for Arch2POCM.

With SGC, we now have nine companies engaged in collaboration with us and funding us. We file no

patents, and we are now collaborating with pharma to generate new chemicals that are put into the public domain to test new ideas about diseases. Four years ago this would have been viewed with amusement, with—I'm making this up—"there goes Aled, talking about a fantasy world in which pharmaceutical companies share chemistry with the world," because chemistry was and in many respects still is the inner sanctum of pharma, where the value is created.

But it has come to pass. And we have evolved a collaborative framework in which the professors and companies collaborate to make these new molecules, and put them in the public domain so everyone can use them. This was one of the key inflection points that allowed us to think about moving the pre-competitive barrier "downstream" to where we believe it is more appropriate.

HASSAN: *So in this pre-competitive collaboration that is SGC, what were the key lessons you learned that made it possible to work with the pharmaceutical companies?*

ALED: I think first of all, you need to be transparent and have a clear mandate. We had credibility because we have never patented anything, and we have a large collaborative network of professors that has worked with us for free and has never filed for IP.

It is also important to choose the right problem—a problem that was perfectly suited for sharing. We chose an area of science called epigenetics that everyone thought, "Wow, that's pretty exciting, but hell, is it risky." The concept was to share resources and expertise, and trust the Consortium to get the job done.

The next argument was that industry only pays 10 cents on the dollar to get data that they could use internally to do whatever they want, including starting a proprietary program. Meanwhile the public benefits from the knowledge going into the public domain.

HASSAN: *That's a win-win. Were there any management or people challenges that were difficult to overcome?*

ALED: Yeah, there were minor hiccups along the way both on the academic and on the pharmaceutical side—people not wanting to share as much as they should, people struggling with what's appropriate to be open and closed. But with hiccups we're learning as we go, and the same thing will happen with Arch2POCM.

The power of the idea and the data that we're generating is enough to convince people to stick with it. And now it's really taken off—it's exciting. For SGC, every company is donating about 8 million dollars of cash and in-kind to a project for which they get no proprietary patents. In total from the pharmaceutical industry we probably have over 60 million dollars in resources over four years. It's a tremendous amount of resource for this important problem.

HASSAN: *Impressive.*

ALED: And we never, ever file for IP in the SGC.

HASSAN: *Aled, let's take a step back and think about collaborative health R&D more broadly. You talked a lot about Arch2POCM and briefly about SGC. Are there any other specific collaborative tools or approaches that you find particularly promising?*

ALED: Any efforts that share knowledge and put it into the public domain are fantastic, like the GWAS (Genome-Wide Association Studies) and genetic studies that put genetic information into the public domain. The cancer genome projects, those kinds of earlier-stage sharing projects are a tremendous

advance.

HASSAN: *If you think about collaborative health R&D as a sector, what kind of catalytic efforts or tools on the part of funders, governments, or toolmakers might help the sector as a whole?*

ALED: I think that much of the collaborative advantages are not paradigm-shifting per se, they merely tear down the walls that we've created over the last 30 years—undoing a problem we created in the first place. We've created a sort of siloed innovation cycle where the universities do X and then biotech does Y, and then pharma does Z. What we really need to do is go back to the future—or forward to the past — when sharing data was more the norm.

The dysfunctional system we now have evolved from a bunch of different drivers that made sense at the time, but don't make sense today. I maintain that there's no real reason that we can't all collaborate on the fundamental science, and then if somebody sees an opportunity, they can advance it commercially.

HASSAN: *To dig into that a little more, we've seen for example how much can happen by virtue of mandates for open access in biomedical literature. Is there a similar type of mandate or rule change that would be simple to describe and yet have the same kind of impact as open access had?*

ALED: I think it's quite difficult, because in open access literature, it's really only the publishing companies that have a stake in the game, right?

When you talk about open access for R&D in general, you're talking to a hell of a lot of interested parties, with a lot more money at stake. You're talking to national governments that want to build local economies. You're talking to universities who are being rewarded and want revenues from their tech-transfer operations. Venture capitalists want to make money on proprietary investments, and pharma wants to make money—so asking everyone to share is far too simplistic an idea. It needs to make business sense first.

So we need to ensure that our open access projects makes business sense. I frame the arguments for SGC or Arch2POCM solely on their economic ROI, even taking into account the caveat that there is a partial free-rider quality for those project non-participants who do not share data and results. Indeed, in terms of the projects that we're trying to accomplish, you can easily make the argument why it's cheaper and more cost-effective to do it our way than by doing it in secret.

HASSAN: *It sounds like it's a matter of finding those new business models that generate both private value and also public value.*

ALED: Exactly. It's got to make both scientific and economic sense.

And for those open access projects that do both, there should be mechanisms to support them from governments. Right now, the true pioneer areas of science have a very difficult time getting public funding because we tend not to be too risky in the public sector. That's why a foundation might be the better way to go—a real risk-taking foundation.

HASSAN: *That's a hopeful vision for the future. Last question for you—and it's a personal question—it's very clear listening to you that you have a lot of passion for many of these ideas. If you look back at the past several years you spent on collaborative health R&D, what really drives you to put your heart and soul into this? What's your passion in this area?*

ALED: I think the disservice that we as scientists are doing society. I feel badly that we can't invent

more medicines for our children, for the people who are sick, because of the ineffective infrastructures that we've put in place that don't serve the common good and that poorly serve individual interests. It’s this terrible sense that we're letting people down by the way we've constructed the world, and we need to change and start doing it properly for everyone's benefit.

HASSAN: *Almost a sense of responsibility?*

ALED: Absolutely—social responsibility. You can think of it in terms of your own family if you want; you can think of it in terms of your country or in terms of the global population. It's the same for everything.

We're spending hundreds of billions of dollars and we're not successful, and it's not because people aren't trying hard. We're trying hard but the reward structures we put in place are counter to the public good, and to economic advancement.

And I'm not completely naive—you can understand how all these systems evolved, why universities think the way they do, why the peer review system works the way it does. No one is malevolent; everyone is acting within the reward structures we have put in place.

And so finding a way to get around those built-up enterprises, to create better reward systems, to find a way for everyone to gain—in essence to figure out how to do it right is a fun challenge. At the end of the day what drives us is that if it works, we will dramatically improve human health all around the world, and that's going to be something I'll be proud to be part of.

HASSAN: *Finding a way to do better.*

ALED: Absolutely.

HASSAN: *Aled, thanks so much for speaking with us today.*

ALED: Yes—thanks a lot.
