# Supplementary material for: Ten Simple Rules for Cultivating Open Science and Collaborative R&D
Source: PLoS Comput Biol. 2013 Sep 26;9(9):e1003244. doi: 10.1371/journal.pcbi.1003244 (PMC3784487; doi:10.1371/journal.pcbi.1003244)
Supplement: Text S5 — A conversation with Benjamin Good, coleader of the Gene Wiki initiative. (PDF) [file pcbi.1003244.s005.pdf]

Insider Views of Collaborative R&D for Health: Q&A with Benjamin Good

March 7, 2012

Benjamin Good is one of the leaders of the Gene Wiki initiative. The Gene Wiki contains more than 10,000 collaboratively written review articles about genes in the human genome, with the ultimate goal of providing comprehensive information about every human gene. It makes use of Wikipedia as a low-cost mass-collaborative platform.

HASSAN: *Ben, how can Gene Wiki help global health R&D?*

BEN: The Gene Wiki benefits global health R&D by providing a unique, gene-specific synthesis of biological knowledge. The knowledge about a particular gene's function is typically dispersed throughout hundreds or even thousands of distinct journal articles that may or may not be open access. By aggregating that distributed knowledge into coherent narratives that are freely accessible, easily discoverable, and supported with links to supporting literature, the Gene Wiki serves to better connect both researchers and interested lay people with knowledge about human genes.

HASSAN: *How do you measure Gene Wiki's success today? And how would you like to measure success in the future?*

BEN: We currently measure success in terms of usage, content growth and quality. As we described in a recent publication, we monitor usage based primarily on page views, content based on the number of words, links and references in each article, and quality using WikiTrust. WikiTrust provides reliable estimates of the quality of wiki articles using algorithms that infer reputation scores for each editor based on their editing history. In the future we will continue to use metrics like these but would also like to assess how much of an impact the Gene Wiki is having in specific domains such as science education.

HASSAN: *It looks like your metrics are quite detailed. Aside from funding, what is the biggest challenge you face with Gene Wiki?*

BEN: As with any open content system, the biggest challenge is really recruiting and maintaining a large population of active contributors. Part of that challenge is the lack of any kind of reward from the scientific community for participation in projects like this. How do you convince an expert on a particular gene to spend their time writing a Wikipedia article about it rather than doing something else that has more tangible career benefits?

HASSAN: *Aside from Gene Wiki, what other collaborative tools or approaches for health R&D do you find promising?*

BEN: I think scientific discovery games like the protein folding game Foldit are hands down the most exciting new approach to collaborative innovation in this space. Games offer the potential to recruit enormous numbers of participants and to engage them for long periods of time. Foldit has had more than 300,000 people participate. That is impressive until you consider that more than 20 million people have signed up to play MineCraft. If a scientific task can successfully be 'gamified', the potential is simply enormous.

HASSAN: *Fascinating. Ben, thank you for speaking with us!*

BEN: Anytime!
