# Supplementary material for: Ten Simple Rules for Cultivating Open Science and Collaborative R&D
Source: PLoS Comput Biol. 2013 Sep 26;9(9):e1003244. doi: 10.1371/journal.pcbi.1003244 (PMC3784487; doi:10.1371/journal.pcbi.1003244)
Supplement: Text S6 — A conversation with Bernard Munos, pharmaceutical innovation thought leader. (PDF) [file pcbi.1003244.s006.pdf]

Insider Views of Collaborative R&D for Health: Q&A with Bernard Munos

May 29, 2012

*Bernard Munos is a veteran of the pharmaceutical industry, the Founder of InnoThink, and a thought leader in transforming the pharmaceutical and biotechnology industries. He is the author of influential papers in Science and Nature, such as "*How to improve R&D productivity: the pharmaceutical industry's grand challenge.*"*

HASSAN: *Bernard, thanks so much for speaking with us today.*

BERNARD: You're welcome! Thanks for having me.

HASSAN: *First question, and I know this is going to be tough for you because your interests are broad: give us in two sentences your area of interest and your focus in collaborative health R&D.*

BERNARD: I'd say for the last 10 years it has been pharmaceutical innovation. Specifically where it comes from, who uses it, and how to get more of it.

HASSAN: *Very succinct—I didn't think you make it in two sentences! (laughter) And could you briefly describe what your own personal role has been?*

BERNARD: I started way back in the mid '90s, when I saw things taking place in the pharmaceutical industry that made me feel uncomfortable. Basically, what I was witnessing was a change of culture, from a culture that had been dominated by creativity to a culture that was essentially dominated by process. And being trained as a scientist, I felt in my guts that this did not bode well—that when it comes to innovation scientists are not at their best when they're doing routine stuff, when they're managing processes.

I came to feel that we were harming the legacy of a century of scientific advances that had delivered tremendous therapeutic breakthroughs. This was an industry spending tens of billions of dollars a year to produce innovation, and yet the industry had not taken the time to develop the tools that it needed to really optimize that investment. As a result of that, there was and still is a massive misallocation of resources towards projects that should never be funded, while others are sitting on a shelf waiting to be translated into something useful.

I thought there was a better way. Unfortunately, I couldn't interest a lot of people in it. But I decided that, regardless, this is something that needed to be done and I would go ahead and do it—in my spare time if I had to. Which is essentially what I did, although I must admit that my company, Lilly, allowed me to pursue and spend time on that problem.

Eventually the research that I did blossomed into several articles that were published in *Nature* and *Science*, which had a significant impact on how the biomedical scientific community saw and understood innovation. And not only the biomedical scientific community but also the stakeholders: the policymakers, the regulators, the universities, the think tanks, the patients' organizations, and so forth.

I think it triggered—much to my surprise because frankly I didn't know going in that this would happen—it triggered a spirited debate about innovation. And I think it helped draw people into it, to the point that we are now addressing it squarely as we should. We're coming up with answers, with

insights, with new models. That's positive. That bodes well, because it is helping us find solutions to what I see as being the triple challenge that the industry faces: we need more innovation, we need better innovation, and we need affordable innovation.

We need to successfully address the triple challenge if we are to secure our future.

HASSAN: *As you say, Bernard, that is almost a grand challenge for biomedical innovation. What would you say are the specific approaches that are in scope when we speak about "open innovation" or "collaborative health R&D"?*

BERNARD: Well, "open innovation" is really a collective term to designate a range of models out there, that are quite different but have one thing in common: they are all network models. Some purport to do drug R&D from soup to nuts, from discovery all the way to approval. Others only aim at doing one slice of that whole chain.

But all together it adds up to what we'd call a new ecology of collaborative research, which has created a market for innovation. If you are a scientist today and you are interested in doing drug R&D, you can basically be a one-man pharmaceutical company. Or you can join forces with a handful of friends, and be a virtual pharmaceutical company. And you can decide to focus on your passion, and everything upstream and downstream from your interest, you can in-source it or outsource it or open-source it.

This was not the case back in the '90s when I started to scratch my head about innovation. I think we've made great progress—much of it enabled by the Internet, needless to say. Both in this country and around the world, we see scientists and organizations today getting into drug innovation that are completely new to that game. You have vibrant open innovation platforms in places like India, for example, with the open source drug R&D platform; in Africa, with the African Network for Drug and Diagnostic Innovation; and soon in Asia and Latin America with similar organizations.

I think we're seeing all kinds of interesting stuff. The more actors we have in innovation, the more successful we will be. The more intellectual diversity and scientific diversity we bring to that game, the more successful we will ultimately be. It is encouraging, but it is also quite different from the way we've done it in the past.

HASSAN: *You mentioned your papers that you have written for Nature and Science, which were quite nice papers that addressed the role of this sort of network innovation for biomedical R&D in general. Could you share your thoughts on the specific value that this sort of collaborative health R&D might have for global health in particular?*

BERNARD: I think the value of network R&D is to help address the third challenge that I mentioned earlier: how to produce affordable innovation. Network research models are very cost-effective; their economics is very compelling. Some of them have been operating for more than a decade now, so we're starting to have good data to look at what they've been doing, and how much money they've spent, and what they have produced.

A good rule of thumb is that they allow cost savings that range from 80 to 98 percent. This is very significant, needless to say—also very disruptive, very transformational, because there is a lot of inertia in the system. But when you have that sort of improvement in efficiency, sooner or later it is going to prevail.

We see that happening. If it takes some time in this country, it's going faster elsewhere where the pressure is higher. This is why you're starting to see some interesting things in places like India and

China and a few others.

Those people want to play the game. They want novel therapies, especially novel therapies for diseases that are not addressed by traditional pharmaceutical companies: neglected diseases, rare diseases and so forth. But they don't have the billions of dollars that it takes to play the game in a traditional way. So they are basically reinventing the game, so that they can play it within their budgets. I think they're reinventing the R&D model in a way that ultimately threatens to be transformational, and to impact the way we're doing it in the major countries as well.

HASSAN: *When you speak about cost savings of 80 to 98 percent, those are very compelling figures. What are a couple of specific examples of that?*

BERNARD: Well one of them—the most compelling—is InnoCentive. Many people are familiar with InnoCentive. It is essentially a problem-solving platform on the Internet that allows scientists or organizations that are facing serious scientific enigmas—in other words problems that they cannot solve themselves, difficult problems—to pose those problems on the Internet, along with a prize that would go to someone who can solve it.

In the case of InnoCentive, they have assembled a community of problem solvers around the world that numbers almost a quarter million people—around 250,000 scientists. When someone posts a problem on InnoCentive, an email goes to each of one of those scientists. As happens often in science, the problem that I have has already been solved by somebody elsewhere in the world—I just don't know who that person is. InnoCentive allows me to find that person and connect with him or her. Sometimes they don't have the solution, but they can find it with very little work.

If you go to the statistics that are posted on the InnoCentive website, the first thing that you see is that it does not work in all cases—it only works in about 50 percent of the cases. In the last 10 to 12 years or so, there have been 1400 such challenges posted on InnoCentive. Half of that, roughly 700, have been solved. For those 700, the amount of prizes that have been paid is around $17 million I believe.

Now if you work for a major pharmaceutical company, and you're trying to tackle one of those tough challenges—for example, if you're trying to find a route to synthesize a compound that is very difficult to synthesize—if you try to do it internally, it's a safe assumption that it's going to take you maybe a couple of years. It would not only involve you, but would also involve some technicians, some assistants, and so forth. So the cost of finding an internal solution is most likely above $1 million.

But let's assume that we're more successful than we expected, and it takes us $1 million to solve that challenge. So 700 times a million is about $700 million, which would be the cost of finding solutions to those challenges if they were handled internally. InnoCentive does it for $17 million.

HASSAN: *That seems compelling.*

BERNARD: Yes, you do the math and the cost savings are enormous. And it's not just the cost of finding a solution—the timeline is also much shorter on InnoCentive, because those solutions by and large have already been formulated somewhere.

So you can see you've got a very powerful platform there that can really do a lot of good to folks who work in drug R&D and elsewhere.

HASSAN: *When you think about the enterprise of collaborative health R&D as a whole—in other words all of these collaborative approaches taken as a category—how would you measure its success*

today?

BERNARD: I think the real proof—what everybody wants to see—is how many novel therapies are coming out of all that. And it's a tough question because developing a new drug as we all know is a lengthy enterprise. You have to do trials, which cannot really be compressed and which must produce compelling data that satisfies the requirements of the regulatory agency. Traditionally it takes five to ten years to do that.

Fortunately we're starting to see some outputs. One public-private partnership—which is another type of network organization that focuses on neglected diseases—is Medicines for Malaria Venture (MMV), which is dedicated to finding better antimalarial drugs. They've been in operation for the last 12 years. And in the last 12 years a handful of people—a couple of dozen—have explored I think over 700 ideas on how to develop new drugs for malaria.

They have funded over 50 of those ideas, and explored dozens of different classes of novel chemistry, and run dozens of clinical trials to test some of those drug candidates. They have registered one drug which is Coartem, and have several more that have been submitted for approval. And they have all kinds of novel therapies in various stages of development, which offer the prospect of successfully dealing with a scourge that has plagued mankind forever.

Now I must give credit to the corporate partners that are helping MMV in that process: companies such as Novartis, GSK and I think Sanofi and even AstraZeneca have been involved in that. Because it does show, number one their farsightedness, and number two their commitments to innovation. It is a game that is best played in a collaborative fashion—leveraging the resources that exist in various organizations, in order to achieve a goal that benefits us all. I think MMV has done it quite successfully, and has done it on a shoestring. MMV's budget annually is still around $50 million, and they're doing all that research for $50 million, and paying their own cost. And all that is done by a few dozen people.

I think it illustrates how far you can go on a shoestring. You don't really need thousands of people to do drug R&D. Some organizations do it with a handful of people, and they do it successfully and inexpensively. We need to learn from those models, and maybe start emulating them, as opposed to insisting upon the traditional way—which is very expensive, very slow, and frankly no longer delivers very exciting things as far as innovation is concerned.

HASSAN: *Hmm. If we were to abstract out from the two examples you mentioned so far of MMV and InnoCentive, one metric that one could perhaps partially quantify would be value for money. A second might be harder to quantify – something like the speed of innovation, or perhaps even probability that you would actually achieve a good solution in some time span. How do you think we might build on these metrics for success, and how could we better measure success in the future?*

BERNARD: Yes, you're right. This actually addresses the triple challenge that I was mentioning earlier. Speed is directly related to the quantity of innovation that is being produced.

I think the metrics as far as the patients are concerned and society is concerned are the same: How much do we put in? How much do we get out? That's what we have got to worry about.

How much do we put in today? Well over $200 billion a year.

What do we get for that? We get basically 20 to 25 new drugs a year—most of them marginal, and most of them not that much better than what's already approved. And few of them address the problems that

we really need to address. Those include rare diseases and neglected diseases and things like that.

So it's not that medical innovation is at risk of running out of money. There is an enormous amount of money that continues to be invested every year in pursuit of innovation. It's just that that money doesn't produce much. That's what we need to address.

HASSAN: *So success would be something like—let's say in five years from now, 10 years from now—if we had a new approach to R&D that used these methods and had come up with a drug or therapies that were clearly superior, and for which success could be clearly attributed back to these methods?*

BERNARD: Yes. And I think we will see that as our new model continues to spread, and as more and more companies get into the act of developing new drugs on a shoestring.

I think we've got things that happened in the last 10 years that are starting to make a difference. And I don't want to give the wrong impression by saying that this is an enterprise that goes against the pharmaceutical industry, because some companies have actually been quite enlightened, and have supported and co-funded and helped those network models to come into being.

Lilly 10 years ago had a division which produced a number of novel business models, some of which have been quite successful. You're talking about InnoCentive, of Collaborative Drug Discovery, of YourEncore and so forth. We mentioned earlier companies such as Novartis or GSK or Sanofi or AstraZeneca helping public partnerships focused on neglected diseases – helping them to succeed. GSK has put a whole facility in Spain, and has opened it to scientists from the third world who want to work on those diseases using GSK resources at that lab. So I think there has been some progress made with the help of the pharmaceutical industry. We just need to see more of it.

HASSAN: *It's certainly a hopeful vision for the future. In order to achieve that hope, what do you see as the biggest challenge facing collaborative health R&D?*

BERNARD: I think even though the industry has done some very commendable things to help out, there's a lack of alignment within each large pharmaceutical company on how to go forward.

I think it's fair to say that in all broad pharmaceutical companies today you've got folks who are advocating and pushing to transform the research model, and to embrace open innovation, and to embrace whatever will produce more and better and affordable innovation—and then you've got folks who will have none of that. That sort of argument can end up being quite dysfunctional.

Those companies in which the top leader, the CEO, acts as a de facto Chief Innovation Officer tend to fare better. Because when the top guy is the boldest of all, it creates a secure environment for the scientists in the organization to do what they should be doing, and that is to think boldly of new ways to come up with breakthrough therapies.

HASSAN: *Right—it sets the tone for the whole enterprise.*

BERNARD: But when the top guy delegates that responsibility to some underling—whether that person reports to him or it's several layers of management below—it does not work as well, because it basically allows the people below to fight it out. That tends to be dysfunctional.

So I think we've got a bit of a leadership problem in the industry. Not in all companies—some companies are blessed to have some very effective leaders that are doing a very impressive job in transforming the organization. But we need more of them.

HASSAN: *Bernard, have you come across an example where a company or organization feels that it*

may have the capacity to solve its challenges internally?

BERNARD: I'm sure some organizations still feel that way, and I'm sure this is being used as an argument to steer away from collaboration, to steer away from network models. Frankly, at this stage I think we've got an ecology of organizations and models out there that is rich enough that in my view the more competition we've got, the healthier it's likely to be.

If some companies mistakenly believe that they're better off doing it all by themselves, the traditional way—the market will eventually sort that out. We've got all kinds of entrepreneurs out there, and all kinds of incumbents who believe that their way is the better way. I'd say let competition play it out, and sort out which of those models are most effective at addressing the triple challenge that we mentioned earlier.

I don't think we should be dogmatic and pretend that there is one solution to the problem—there is not. I think there are multiple solutions to the problem. Some are better than others, and some are maybe more suited to certain scientific challenges than others, but ultimately I think all that will get sorted out.

We're going to go forward from here, and it's only going to get better.

HASSAN: *Speaking of going forward, which specific tools or approaches for collaborative health R&D do you find promising, especially emerging or less popular ones?*

BERNARD: Well, if you look at what's out there already, it seems that computerized platforms seem to be able to deliver lots of value for various reasons—one of which is that a good rule of thumb is that whenever you can take something from a wet lab and bring it *in silico*, you basically eliminate 90 percent of the cost and 90 percent of the time. It's a rule of thumb but I think it's roughly accurate.

HASSAN: *An order of magnitude improvement.*

BERNARD: That is a tremendous improvement in productivity—but there is more than that. Computerized network models bring together people from all over the world. They're really very effective at tapping the global brain. And as I mentioned earlier, whenever you do that you avail yourself of solutions that could not have hatched into your own brain because it wasn't wired the proper way.

Basic differences lead scientists to approach problems in very different fashions. If you can leverage those differences and exploit the synergy between them, research has shown that you can do a great deal of good as far as innovation is concerned. That's one of the benefits of those computerized platforms—everybody can sign in and everybody can interact with people who are coming to the same problem from a radically different perspective. And we all enrich each other's insights and do it in real time 24/7, which is something that in the past was basically impossible to do in closed organizations.

The more those models spread and keep taking root, the more the triple benefits that I mentioned earlier—faster speed, lower cost and higher quality, and shorter timelines—the more of that we're going to see.

HASSAN: *It sounds almost like replicating the flowering of creativity that happened with the Internet, but we're doing it now specifically for collaborative health R&D. It's fascinating to think forward about what's going to happen.*

Bernard, a couple of last questions as we move toward the end of our conversation. What do you think might help to guide interested people or interested parties to better use collaborative health R&D?

BERNARD: Passion is I think an essential ingredient there.

A lot of people, especially scientists, decided to become pharmaceutical scientists because they were passionate about science and they wanted to do some good. Many of them were passionate about doing some good. Unfortunately the cultural change that swept the industry 10, 15 years ago that I alluded to earlier in our discussion—that "process culture" that is now dominating the industry—has basically rooted out the passion that needs to be there in order for innovation to flourish. And so I think we need to bring the passion back to what we do at all levels.

HASSAN: *A culture of passion.*

BERNARD: That's right, which the industry has had for most of its history. It's in our DNA—we just need to express it again.

I'm optimistic. Even though we face a severe innovation crisis today, I think biomedical innovation will survive and will continue to thrive, and it will only get better. But who will do it will depend upon the battle that we're currently seeing taking place between the incumbents and the insurgents.

Traditionally, if you look at the history of disruption in other industries, we can draw some conclusions because the blueprint is always the same. What typically happens is that industries survive disruption, but the incumbents seldom do. Those companies that survive are those that manage to redesign their business model in a way that overcomes the challenges.

To bring it back to the pharmaceutical industry, I think the incumbents today can do that and bounce back like Apple did, or like IBM did—or they can wither like Kodak did. And I think the difference will be made by the leadership of those organizations.

HASSAN: *In order to achieve the disruptive innovation you're talking about and to help more people become Apples and IBMs as it were, what low-hanging fruit do you see in terms of supportive innovation or tools that might help these kinds of network health R&D approaches become better used?*

For example, things like a collective roadmap or conferences or metrics or a group blog. In your view, what do you see as two or three examples of really low-hanging fruit, which should be done sooner rather than later and are easy to do?

BERNARD: Well, I think all forms of collaboration are good and will help, so that needs to be encouraged. Collaborating is not really an expensive thing to do and it's one that does deliver. So it begs the question, why don't we see more of it? That's one thing.

The second thing is that I think we need to open our minds to new things. It's not always easy to do, but there are all kinds of new technologies out there, that I think can play an important role in improving healthcare around the world, and especially global health.

For example, there are hundreds if not thousands of apps that have been created to do all kinds of stuff. Eric Topol was talking recently about one such app that allows you to do an electrocardiogram with an iPhone, into which you plug in a couple of additional pads. In many countries of the world you have cell phones, smart phones, even if you have very little of anything else. I think nobody has yet had the time to really figure out what we've got there, and how we can harness it in order to help advance healthcare around the world.

I was talking recently with someone at NIH about some entrepreneurs developing video games as a novel therapy for schizophrenia. It takes as we know a few months to put together a video game, versus

10 years to come up with a new drug. You can see how the economics of this can be pretty disruptive, and video games are not regulated by the FDA and so forth.

So you've got all kinds of innovation that is coming in from unlikely corners, from outside the system. We need to do more thinking about how we can string this together in order to improve biomedical innovation and healthcare. I think the ingredients are being produced faster than we can figure out how to use them—but I'm sure that given a couple of years, people like you will start to figure that out. It's going to have an important impact on all of us.

HASSAN: *Absolutely—it's a challenge for all of us. It's fascinating how as you've spoken today, you're often speaking about a culture of passion and opening your mind. It seems like both some of the barriers and also some of the best opportunities are in our own approaches to the problems, as opposed to in a technical solution.*

BERNARD: Yes, this is correct. We need to stop the blame game which I hear all the time going around the industry, "It's not our fault, it's somebody else's fault." No – I think we've got the resources within to deal with our problem and we should focus on that.

If a company today cannot produce innovation or cannot produce enough innovation to secure its future, it's its own fault. It's nobody else's fault. While the industry has declined in the last 10, 15 years, some companies—admittedly a minority of them, but some companies such as Genentech—have done extremely well. What could a company such as Genentech do that the rest of the industry couldn't do? I could multiply the examples, but the excuses aren't helping.

I think we've made mistakes. We've got to acknowledge that and we've got to learn from our mistakes and humbly move forward, and we're not always doing that as much as we should.

HASSAN: *I hope we'll do more of it in the future. Bernard, last question, circling back to the start of our conversation when you spoke about what brought you to this place in your life: what keeps you going? What's your passion in this area?*

BERNARD: It's innovation. It's a topic we'll always be interested in. I think it's a smarter way frankly to create decisive competitive advantage.

I was trained as a scientist. I was also trained as an economist and as a businessman. The creation of competitive advantage is a fascinating business problem. You can do it in two ways; you can do it by trying to optimize and improve the performance of the existing system, or you can do it by changing the rules of the game. And I always felt that the latter was more fun but also more effective.

Steve Jobs has a famous quote when he remarked that "it's a lot more fun to be a pirate than to join the Navy" (laughter) and I think there's a grain of truth there. Large companies need to allow more of their employees to be pirates, just like Apple tried to do and ultimately did.

HASSAN: *Well, that's an unusual and inspiring analogy! Bernard, thank you so much for speaking with us.*

BERNARD: You're welcome. Thanks for having me.
