# Supplementary material for: Ten Simple Rules for Cultivating Open Science and Collaborative R&D
Source: PLoS Comput Biol. 2013 Sep 26;9(9):e1003244. doi: 10.1371/journal.pcbi.1003244 (PMC3784487; doi:10.1371/journal.pcbi.1003244)
Supplement: Text S7 — A conversation with Zakir Thomas, director of India's Open Source Drug Discovery (OSDD) project. (PDF) [file pcbi.1003244.s007.pdf]

Insider Views of Collaborative R&D for Health: Q&A with Zakir Thomas

August 2, 2012

Zakir Thomas is the Project Director of Open Source Drug Discovery (OSDD). *Based in India, OSDD has attracted over 6000 contributors. Focused on drug discovery for tuberculosis, it has recently begun* investigating malaria *as well.*

HASSAN: *To start off, what's the big problem that you're trying to solve with OSDD?*

ZAKIR: The big problem we are addressing is to get new drugs to the market. We started with tuberculosis, and that remains our first target, but we decided to extend this to malaria.

That challenge is complicated by the fact that we are dealing with a neglected disease—I prefer the term "disease without market." In such a situation, we need an alternative model of innovation. The challenge is to create that alternative model so that drugs reach the patients who need it most.

HASSAN: *If you were to summarize how OSDD works in a nutshell, what are the main components of this new model?*

ZAKIR: OSDD is a collaborative approach to bring together the best minds we can on a platform where people can remain in their core competencies, and contribute through the drug discovery pipeline.

These contributions form an assembly line of collaboration and contributions stitched together by this collaborative community effort, managed with the funds of OSDD provided by the government of India. Pushing the drug discovery process down the pipeline, furthering innovation at all stages while keeping the cost at minimum—that's what we do.

Researchers, serious scientists, students who could contribute, or even social scientists who would like to simply comment and study how we do all this—all these persons form part of the OSDD community.

HASSAN: *Let's dig into that a little bit. I understand how the collaborative platform works if the contributions are more informational in nature. How do you handle it when you are looking for contributions of wet-lab work or more physical work?*

ZAKIR: See, the work that is going on in the wet-lab has two components: the actual experiment that takes place, and the information that gets generated from it (which will either be published or patented). That's how science works. And in the case of an open-source program, especially in a drug discovery program, you need to have serious wet-lab activities.

HASSAN: *Yes*.

ZAKIR: There are serious researchers who have wet-lab facilities doing wet work in TB, who have shown interest in working on OSDD. They submit their project, and then there is a process of community approval—it's approved, funded. They do the experiment, post the research data back, and the research, if it is encouraging, is carried forward.

I'll give you one example. There is a target which was identified by a bioinformatics group at the Institute of Genomics and Integrative Biology at Delhi. GlmU is the target name. A group of wet-lab

biologists at the National Institute of Immunology in Delhi developed assays for GlmU.

Once you have the assays, you need the chemical components to be screened. These components were developed by academicians at universities in Gujarat and Hyderabad. So these guys synthesize the compound, send it to the National Institute of Immunology which screens them, and the research goes there and back. You can see where an informatics component is linked with the biology wet-lab work and linked again with the chemistry work—a chain is set up and working.

HASSAN: *How do you handle the issue of quality control?*

ZAKIR: Like any other scientific project. When you go to the OSDD website, you see a link called OSDD Chem. This is collaborative chemical library generation, where a large number of university participants are synthesizing molecules, sending it to a central repository, and that central repository is screened against Mtb (Mycobacterium tuberculosis).

In that process, it's insisted that they all upload the HPLC (High-Performance Liquid Chromatography) data, which is not normally asked even for publications from academic institutions. But OSDD asks the investigators to upload the HPLC data online so the whole community sees what the purity is. And if the purity is not up to the desired level, then this won't be taken forward.

HASSAN: *One of the issues that people have raised about these sorts of collaborative endeavors is: if you have a large set of partners and institutions, how do you generate trust and assess how good each contributor is?*

ZAKIR: In science, I think the biggest assurance of quality and trust is peer acceptance. That's what the open-source platform enables. You post research online, which is seen by peers.

Before submitting his research results, a scientist who is serious about his career as most scientists are, would be serious about his reputation among his peers.

HASSAN: *Yes.*

ZAKIR: That is the mechanism that we are using to ensure quality control. Just use the existing forces that assure quality control in science.

HASSAN: *Okay. Let's take a step back and look at OSDD from a more strategic level. If you think about OSDD as a whole over the next two, three, five years, how do you see OSDD helping health, specifically in the area of global health or neglected disease R&D?*

ZAKIR: We are moving toward conducting clinical trials of a molecule this year. It's not internally developed by OSDD, but through a collaboration with the TB Alliance. It is subjected to the OSDD principle that it would be available in India to TB patients without any royalty, without any intellectual property encumbrance.

We will be doing clinical trials in more than one molecule in the next two to three years' time. The clinical trials will be funded by OSDD, and will be managed by OSDD. The main concern of industry is how to de-risk the clinical trial, which is very expensive, particularly when conducted in developed countries. OSDD's way of de-risking is funding the clinical trials. If the molecule comes through the trial, make it available to the patient. If it does not come through, we take the risk.

We should also, in the next three to five years' time, be doing activities that improve the scientific understanding of the bacteria that causes TB. One major problem in most of these diseases is the lack of understanding of the pathogen which causes the disease. If I portray the drug discovery funnel, the

top of the funnel has to be broadened so that you have much more coming down at the end of the funnel.

Our effort will be to increase the scientific understanding of the host and the pathogen and its interaction, synthesize a large number of molecules for tuberculosis screening, and then do extensive wet-lab activities identifying targets. We will then take those early hits to leads and then forward through the drug discovery pipeline.

HASSAN: *Looking at OSDD's success or metrics for what OSDD has accomplished to date, what are the key metrics you use to measure or publicize OSDD's success?*

ZAKIR: It's a very difficult question to answer, because most of what we do, most of the time, is completely uncharted—new areas, new challenges.

When this project was initiated, there were a lot of naysayers who said it will never work—drug discovery cannot happen in an open source way.

From that kind of negative image, from a period of complete disbelief of its viability, we've taken it to a stage where it is nowadays seen by many as a serious initiative which needs to be watched.

HASSAN: *When you say it's a serious initiative, the ways you would demonstrate that might, for example, be you have over 6,000 people who are taking part and you have some publications in reputable journals—that kind of thing?*

ZAKIR: Yes. In any such open-source or scientific project, to have 6,000 people registering is a major reflection of the interest of so many from around the world. The publications reflect the scientific activity that goes on.

Ultimately, success will definitely be whether we will be able to deliver a drug to the market, at affordable cost. There is no doubt of that. But the fact that we've been able to put a translational platform into operation is a considerable achievement. This is essentially what a pharmaceutical enterprise does.

HASSAN: *It's certainly groundbreaking. Looking at that platform where you have these thousands of people taking part, in your experience what are the key motivations for people to take part? And how do you handle credit if, for example, there are five or 10 or 50 people who contributed to a specific advance?*

ZAKIR: What motivates people to join OSDD is the broad objective. When you're working on a disease where a lot of people are dying, where the drugs that are being administered are from the '50s and '60s—that is a real motivation for people to come and join and work.

There are people who are coming with very serious scientific work done over several years, contributing molecules on to the OSDD platform. This is done because they believe that OSDD works as a translational platform and is capable of taking it down the drug discovery pipeline.

But we can hold them and retain the industrial people in a scientific project only if we assure that everybody gets due credit for the work they do. If you make a comment today on the portal, it will be time-stamped and date-stamped against your name.

And the scientific discipline is that if you do not attribute and you copy that without attribution—for a scientist, I think peers will very seriously look down upon any such activity. Plagiarism is a charge which no scientist would like to face.

You also asked me what we would do if 50 people contribute?

HASSAN: *Yes.*

ZAKIR: There is a paper that is getting published on PLOS ONE which has more than 50 authors. The paper's abstract is posted online so that everybody can see who the authors are.

Anybody who feels that his authorship is not there can always come up online and say, "Look, I also contributed, but my name is not there," so that somebody can take care of it.

HASSAN: *Interesting. It's almost moving toward the experimental particle physics model, where you have hundreds or even thousands of authors who have contributed.*

In terms of the challenges you face with OSDD, money is always one—but aside from money, what is the biggest challenge which you currently face at OSDD?

ZAKIR: In fact, money is not the challenge. The biggest challenge is the mindset.

Scientists are individuals with different backgrounds. Collaboration and working together, particularly in an area where patents could be taken—to share research and results in the open, this is still not easy. You have to sit, talk to people, convince them.

There are many who come forward. But convincing outsiders who have not contributed to OSDD, who doubt whether an open-source model would work, is still difficult. I think mindset challenges are much, much bigger than any other challenges.

We need committed people and more scientists. Prof S K Brahmachari, Chief Mentor of OSDD who initiated this movement keeps stressing on the emotional involvement of the OSDD community as a significant force that gives it momentum. So far the results have been encouraging.

Dr. T. Balganesh, who has been heading AstraZeneca's research in Bangalore and leading their tuberculosis activity for several years, has joined OSDD. He's a Distinguished Scientist, the highest scientific position that is available in India. The very fact that he left his job in AstraZeneca and joined OSDD shows that money is not the motivation, because he must have been earning many, many times the salary which he would otherwise earn here. The motivation is—according to him, when I discussed with him—the opportunity to develop a new model of drug discovery and the commitment to take new TB drugs to the patients.

HASSAN: *So it's really about winning hearts and minds. As you were talking, I was thinking about the challenges that open access publication faced five or 10 years ago. Once people saw that it was a new model that was achieving a lot, then a lot of people came on board. I think you're probably facing the same challenge over the next five years or so.*

ZAKIR: As you said about open access five years ago, collaborative health R&D has come to stay. It is probably the way of the future for a pharmaceutical industry with drying pipelines.

HASSAN: *Of course, every advance has challenges. If you think about all these collaborative health R&D efforts along with OSDD, are there any catalytic investments you see—aside from money—which you think could help all of these initiatives work better or achieve more?*

ZAKIR: Funds are important. But that is a must and is a basic requirement, so we keep it aside.

Besides funds, there are a number of other challenges. One is that there are significant management challenges in open innovation and the OSDD model. Managing an open innovation project is a

different cup of tea than managing in a closed innovation model.

See, in any pharmaceutical company or research laboratory, there are a group of people who you are directly controlling. You can get work from them. In an open innovation model or an open source model, you need to motivate people. You need to excite them to work with you and to work towards a common goal.

That requires a different managerial ability, approach, and competence. And that is a challenge which is not easy, and it's a resource that is not easy to find.

We have a large number of investigators working with us. There are people who are very good at working with large communities, large numbers of people. But there are serious, brilliant scientists who are not comfortable working with large groups of people. So there are management challenges in locating and identifying people, and letting them lead the team to address their challenge and work together.

HASSAN: *I've heard this from several people before, and it's also a commonly cited challenge in large-scale open-source software projects. What specific help or resource might help to overcome that managerial challenge?*

ZAKIR: Well, I hope training would bridge it to a large extent. But you need to have people who like to do that kind of work – the right psychological bent of mind to work in larger groups and larger communities. It's a management challenge.

HASSAN: *I think articulating that challenge is helpful. Last question to close the interview: on a personal level, Zakir, you've obviously invested a number of the best years of your life into this project. Tell us what motivates you.*

ZAKIR: What motivates me is to get drugs for tuberculosis patients on the open source platform. I love to work in large teams, and I'm a team player. I believe that the Internet provides us unparalleled opportunities, which did not exist before.

I hail from a very rural background in India, and grew up in a world which was not networked the way it is networked now. We didn't have opportunities to meet the outside world. So in every aspect of OSDD, we try to bring together mechanisms which enable people to break out of their constraints and the limitations of their physical existence—to collaborate with people beyond their limitations, and do something which they couldn't otherwise do.

That is what excites me—that it enables us to drive a project which gives opportunities to a large number of people.

HASSAN: *Inspirational. Zakir, thank you so much for speaking with us today!*
