# Supplementary material for: Ten Simple Rules for Cultivating Open Science and Collaborative R&D
Source: PLoS Comput Biol. 2013 Sep 26;9(9):e1003244. doi: 10.1371/journal.pcbi.1003244 (PMC3784487; doi:10.1371/journal.pcbi.1003244)
Supplement: Text S8 — A conversation with Matt Todd, open science and drug development pioneer. (PDF) [file pcbi.1003244.s008.pdf]

Insider Views of Collaborative R&D for Health: Q&A with Matt Todd

September 13, 2012

Matt Todd is an open science pioneer at the University of Sydney in Australia. His previous open science project *produced an improved drug for schistosomiasis. His current project is focused on open source drug discovery for malaria.*

HASSAN: *Matt, to start off, tell us: what's the big problem that you're trying to solve?*

MATT: To make the process of science research more efficient. We're hoping to do that by making the Internet into a collaborative medium rather than just an information resource. We would like to lower the barriers to participation in real research problems so that anybody can participate, and that requires the use of the Web as a place to do science.

HASSAN: *That's a worthy challenge. Before you get to how that might happen, please give us a bit of history.*

MATT: Our first project was on the Synaptic Leap, a website started for the purpose of making biomedical research more open. The problem we posted was a problem which we didn't think that we could solve on our own–we were looking at a problem in public health which needed to be solved, but with a very serious price constraint on the solution.

Crucially, we didn't know who could solve the problem with us. It's a common situation in science: you don't know who to collaborate with, but you know that you need to collaborate.

I had a sense that I knew how to solve this problem, but not within a reasonable price constraint–and yet I knew that there were a whole load of people out there who were academics or industry guys or even policy people who could chime in and get the project back on a realistic track.

HASSAN: *What was the problem?*

MATT: To improve a drug used to treat schistosomiasis or Bilharzia. We started the project with all kinds of fancy ideas about how we were going to solve it. Because the problem was openly available on the Web and everyone could read exactly what we were doing day-to-day, and because we put a bit of effort into publicizing it, people started to notice what we were doing.

We got a gradual trickle of advice from people we didn't know saying that we were going about it in a kind of rarified, academic way, and that we should change the way the project was going.

HASSAN: *When was this?*

MATT: This was early 2010. And so because of that advice, we abandoned one approach and started on another one–but this new approach involved techniques we didn't have a lot of experience with. We got to a certain point where we hit a roadblock and didn't know what to do.

We put out an appeal openly on the Web. In various venues, we asked for assistance with a specific technical point.

Industry really stepped up. They suggested solutions. Ultimately, crucially, a contract research organization in the Netherlands volunteered to do some work for the project for free which would get

us over this roadblock.

HASSAN: *Why do you think they helped you?*

MATT: That's a very interesting question. These guys work in a competitive environment, and yet they were spending time and resources helping the project.

There are a number of reasons. One is that it was a philanthropically valuable endeavor. Another is that people like to solve problems, of whatever kind.

But a further reason is that the company could talk in detail about what they're doing, because it's an open project and there's no IP (intellectual property). Openly on the Internet, they demonstrated clearly in real time how effective they are at solving problems. So if you read about this project and think, “I've got something just like that that I need help with,” you're going to go to this company because they can solve it quickly. And of course they ultimately were named as co-authors on a publication. It's a very effective demonstration of their core competence.

HASSAN: *What's happened since then?*

MATT: That solution was taken back in by us, and we optimized it and developed a process which is now sufficiently promising that the World Health Organization with its collaborators have taken it on for proof of concept studies. We did this on a gram scale, but it needs to go to a ton scale.

Unfortunately, now the project is going to be concealed from public view because we don't control it any more. But the work that we've done is out there and can be used by anybody for any purpose, as long as we're cited. So any company that's interested in making this compound for any purpose can just go ahead and do it without investing any money in R&D.

HASSAN: *It sounds like the model is that the initial steps were funded by grant money–that was out in the open, with some help from a company as you described. Then once you got to the stage where it was closer to market, it went private because organizations needed to make back their investments. Is that correct?*

MATT: Well, we had grant money to solve a certain problem, and then we passed on the results to others who had no contractual obligation to work in an open source manner. It's not particularly surprising or a problem–it's just that different people are now doing the work.

HASSAN: *Pushing that a bit further, how far do you think one could go in the drug development process while keeping things open?*

MATT: I think there is value in doing things open source when you're attempting to solve a problem, because more minds on a problem make the research go faster. If you get involved in a fairly well-worn process, then the advantages of being open diminish.

Let me give you an example. Another big open project was spawned from this first one, which is on open source drug discovery where we're trying to find new compounds for malaria.

The advantage of open source in the malaria project is that we are trying to discover new compounds. That involves a great deal of effort, and it's not clear how to do that–how to solve the problem, what compounds to make, how to go about it. Collaborating with a lot of people there is going to be very powerful.

When you get to the point of deciding on a compound, actually putting it into clinical trials, then there's

not so much advantage in making that process open source because there's a certain process that needs to be gone through, which you can't deviate very far from.

However, it's very important in terms of drug discovery to have clinical trials that are open.

HASSAN: *What exactly do you mean by open clinical trials? What's open, and how much, and for who?*

MATT: The data about how well the compound performs in people, and the monitoring of side effects, for example.

Participants would have to be honest and there would have to be legal consent for people to release data. But imagine that that was all taken care of and you release the data about how well it performed in people. Then there would be no possibility of anybody putting a positive spin on the performance of the compound for any financial reward.

There's so much data that is generated from clinical trials, it's so complicated–in my view, there is no other way of doing great clinical trials unless those data are fully available and fully open. That way anybody can analyze the data and spot problematic trends, or spot issues with the compound, or suggest experiments that need to be done to validate the compound better.

Another crucial point is that clinical trials today treat people as groups, but we're beginning to realize that there's no such thing as a disease on its own – there's only a disease-patient interaction. When you start talking about the volume of data needed to access that, there's no way of dealing with this unless you make those data fully and openly available and searchable, so you can let machines loose on these data and they can spot trends and patterns.

I think this is the future. We're not there yet, but that's going to be exciting in the next few years.

HASSAN: *Absolutely. Returning from the future back to the present, tell us about the malaria side of things with your team.*

MATT: We wanted to demonstrate that we could do a drug discovery project fully open. This is nothing to do with "open innovation", where a company puts out a tender for ideas and people contribute ideas, and the company selects one idea to invest in. This is where everything that you do is fully open. You do work and you deposit it and you work together in a kind of brutal, meritocratic regime where the most effective people are the ones who are doing the work.

You start with a compound, which is known to be reasonably effective at killing the malaria parasite. You take that compound, you allow anyone to make versions of that molecule, and then you evaluate them on how good they are at killing the parasites. You make molecules, you evaluate them, and you change the molecule to make it better.

So we wondered if we could do that open source, where you completely abandon any intellectual property protection on the molecules. That means you can work openly. You can do an experiment in the lab and put it on the Web the same day, and allow people to interrogate the data. You allow strangers who you do not know to help direct the course of the research depending on their competency.

HASSAN: *So everything you're doing is available more or less immediately.*

MATT: Yes. To me, open science is only open if all of the data are freely available.

I was giving a talk about the previous schistosomiasis project, and the chief scientific officer of the Medicines for Malaria Venture was in the audience, a guy called Tim Wells, and we got to talking. And he said, "What would you need to try out a project in open-source drug discovery?" And I said, "Well, you just need some compounds which are known to be pretty good, which no one else is working on in secret because that would duplicate resources. We could start it and anybody could join in."

And he said, "Well, it turns out that GlaxoSmithKline, Novartis and St Jude's just put an enormous number of new molecules into the public domain which are known to be really good at killing the malaria parasite. Why don't we begin a medicinal chemistry campaign to try and develop some of those?" It was the perfect idea.

We've been going for about a year now, and we've got some absolutely sensational compounds, which are really active at killing not just the parasite but the parasite when it's inside a cell. We very rapidly got to a point where the compounds were looking really good.

Unfortunately, recently we tested them orally in mice, which is a late stage usually in the drug discovery process, and these compounds which were super effective at killing the malaria parasite turned out not to be effective when you give them orally to mice. We're trying to work out why they don't work in mice, and we're trying to change the compounds to make them more effective.

HASSAN: *How many people are involved?*

MATT: In my lab here in Sydney, there's me and four other people in the lab. There's a chemical synthesis lab in India, which is contributing compounds. There are three other labs in Australia and one lab in Spain, which are doing biological evaluation. And a few other people have contributed advice on a regular basis online. So all in, at the moment, it's about 20 people.

HASSAN: *What kind of challenges have you had in managing the process itself?*

MATT: Most of my time at the moment is spent managing the process. There are many reasons why people might be nervous about taking part in a project like this. The most obvious is that it's like live TV versus recorded TV. If you make a mistake, it's a public mistake, and it's forever there.

In science we traditionally tend to work privately in our lab. We make local mistakes and it's amusing and we laugh about it over a beer, but it's not public. We publish a paper with a small selection of the data we collected. It makes a paper more like a press release than what we've actually done.

In the open science way, your lab book is on the web and everything you do is there. At some point you say, "Okay, we've done enough experiments. We've got a bunch of negative data, we've got a bunch of positive data; we're going to distill out of that a conclusion, and we're going to publish it." That's what we did with the schistosomiasis project, and that's what we're currently doing with the malaria project.

Discussion about the project is also online–hypotheses, ideas, half-formed sketches of things. I and my students here enjoy the process of doing science in real time publicly–it just comes naturally. But a lot of people are not happy with it, so part of the process is to encourage and reassure and to structure the project in a way that makes people who want to contribute able to contribute without compromising on the openness of the project.

HASSAN: *What advice would you give to someone else doing a similar project in the future?*

MATT: I guess the main thing is to relax [laughter].

Science is not an output–science is a process. We should be treating science more like a software

developer treats a beta product–something that can be released on the Web which is half-formed, and gradually it will be optimized and improved, maybe proven wrong.

HASSAN: *How do you ensure that people get appropriate reward or credit in that kind of process, especially when they're not the first or second author on the paper?*

MATT: That's a great question, because that's key to the whole thing. The way this works in software development is that people who are productive and effective and who make valuable contributions are recognized quite effectively by their community. Gradually, you build up an idea of who's responsible for what.

In science we don't tend to do that so much. We judge someone by their publication record. I think that is going to weaken–there will be other things which will become very important. If a student does a particularly good experiment, that will become recognized by people in the field who then cite that experiment as being the first of its kind, or a particularly beautiful example of an experiment. We'll have data sets which are cited, as well as papers.

I also think it's very important to be clear and open with giving people acclaim for valuable contributions. The contributions are of different types, such as taking part in an online debate or discussion, which can steer a project in a different way. It's going to be perfectly possible for papers describing open projects to name people who have not actually done any experiments, but who are valued and acknowledged for intellectual contributions–and that's absolutely fine.

HASSAN: *More like a movie credit model.*

MATT: Hopefully we don't have a list of contributors that takes five minutes to read [laughter].

Actually, I think it's unusual that still in chemistry, maybe even in drug discovery and most of biology, we have these little papers with between two and five people on them. Other fields are happy with the idea of massively collaborative projects, such as some of the big astrophysics projects. People are quite relaxed about extremely long author lists.

HASSAN: *On that idea of credit and evaluation, let's say you were to take a step back and look at your own project dispassionately with a third-party eye. Obviously if you get a new and better drug at the end of the process, that's a clear win. In the interim, how would you describe or measure your success?*

MATT: Well, that's interesting. The win for us would be that during the course of the project we succeeded in getting a novel compound into phase 1 clinical trials, and I think that's probably our aim for the malaria project. But there are a significant number of things that we want to do before that goal where we would say that yes, we've had some success.

In the case of the previous schistosomiasis project, the key moment when I said, "wow, this is really working," was when the project was accelerated by people that we did not know. That was key. That's why the openness was effective: people could see what we were doing, and that accelerated the research. And that, for us, showed that there's a mercenary self-advantage in doing things openly because your research will be faster.

We want to demonstrate that the same thing can happen with the malaria drug discovery project as well. A major thing for us has been that another lab overseas is making compounds as part of the project. We want to push that much harder–for example, if we are able to incentivize a commercial research organization to make a compound that the project needs. That would be a major event, to get

that kind of input from specialists who are willing to put aside a few hours or a few days of their time to make a compound.

HASSAN: *What's interesting is that everything you mentioned is measuring either the quality or quantity of contributions from other parties, which you can imagine leading to other metrics in the future.*

MATT: Yes, that's right. It's interesting because we've already had a number of things where great advice has come in on the malaria project even when we didn't expect it–solid, sound contributions which we didn't ask for, which have moved the project on.

One spectacular example was a guy who worked for ChEMBL, which is the European molecular biology laboratory database of bioactive compounds based in Cambridge in the UK. A guy who was working for that organization called Iain Wallace did a significant *in silico* (on a computer) analysis of the compounds that we were using in the malaria project, and on the basis of that analysis came up with a prediction of what the compounds might be doing. It was a beautiful piece of work. It was done without us asking–it was done because he was interested in looking at that problem.

There's another collaborator who has stepped in and who is now evaluating that in the lab. That, to me, is the way science should be done. It's a beautiful process, and it happens and it works because everything is openly available.

HASSAN: *Wow, it's like magic mass collaborative genies [laughter]. Tell us about the biggest challenge that you face.*

MATT: I think the biggest challenge is in convincing people to step off the side of the boat–to bare their scientific souls by putting everything openly available on the Web.

In many cases, people are prevented from working openly by psychological barriers. But there's also the institutional barriers. Because of the way in which we are assessed as scientists–on number of papers and their impact factor–people are obsessive about protecting that. They worry that if they put that data openly on the Web that people will steal it or they won't be able to publish it…both of which are not necessarily true, but it's those barriers.

It's the worry of maintaining a competitive advantage that makes people worry about contributing data. And people think that the competitive advantage comes from secrecy. Trying to convince people that that is not true is the most difficult thing.

HASSAN: *Projects like yours and* Open Source Drug Discovery *might be examples showing a different way. On that note, what do you see as the key opportunity for this kind of approach–both for health R&D in general and for global health and neglected diseases in particular?*

MATT: That's a good question. My main thought about open science is that the research is faster and more effective. It is faster because you allow experts to identify themselves, and it's more effective because it is continually peer-reviewed. It's also more effective because all the data are fully available. As time goes by and we build up more and more data, we're going to spot links and patterns between things that we didn't expect.

Now, that's particularly important for neglected diseases, but I think it's important for medicine in general. We are at the moment being outclassed by Alzheimer's and the biochemical chaos that is cancer, and we are just not up to the task because we are still working ineffectively. We are working in

silos. We're possibly more worried about publishing a paper than we are about solving the problem.

We've got to really step up our efforts, and I think that's only going to happen when we as human beings can collaborate without these barriers that we put in place.

HASSAN: *It's a hopeful vision, and yet, I think, a practical one. Moving to the last couple of questions, are there any specific collaborative tools for health R&D, which you find to be promising?*

MATT: We're using a range of things depending on what they're good for. We have the formal lab notebooks, we have blogs which summarize things, and then we have more ephemeral things for publicity, such as Google Plus and Facebook and Twitter.

One problem is that under the hood, a lot of the stuff that we do isn't machine-readable. For a lot of the lab books that we maintain, we're writing in English and using pictures, but much of that can't be properly understood by a computer yet. That's a technical issue, which we're spending time to try and solve–but it's not easy.

The other thing is a psychological thing. I give talks and have conversations about open science quite a bit, and it gives the impression that the project is mine. That's not the case. The problem and the solution are much more important than I am, and we want other people to take leadership meritocratically–yet people who want to contribute feel that everything has to go through me. Again, that's just not the case.

HASSAN: *You're a bottleneck.*

MATT: Yes, and it's exactly the same bottleneck that happened with Linux, the open source operating system. I think its founder Linus Torvalds had to work very hard to democratize the process and make sure that not everything had to go through him. As someone said, "Linus doesn't scale"–meaning that if you try to funnel everything through one person, everything falls apart.

HASSAN: *Following up on those two challenges, are there any catalytic investments you see–such as better tools or better support processes–that you think would help both those challenges and also collaborative health R&D as a sector?*

MATT: There are really, really exciting things happening at the moment in the UK and in Europe. There are mandates for making data more open. These tend to focus on open access to publications, and that's very useful and very important.

I'm hoping that once that battle is won in a couple of years and we go completely open access, that we can focus on the important thing, which for me is that the data behind the papers and therefore that the process of research itself is more open. If everyone in the world had an open online electronic lab notebook that anybody could read, we would be playing a completely different game. Science itself would be a more extraordinary process than it is already.

I think to get to that point we have to have mandates coming from way up high. We need funders, like the Gates Foundation and governments, to say, "Look, we're going to fund you, but we want to make sure everything you do goes public." And then we'll see the changes.

HASSAN: *You've certainly thrown down the gauntlet. Last question is a personal question for you: what personal lesson have you learned in doing open science?*

MATT: It's got me excited about science on a whole different level. As a kid, I loved the idea of science, as we all do when we're kids–dinosaurs and space. It's a fascination that is not impeded by

day-to-day difficulties about how science is done. We love science because we can ask the cool questions and we can do stuff.

Then you grow up and you start a PhD and you start working, and you realize there are a lot of politics, difficulties and barriers behind it. You have to think about lots of other stuff.

Working publicly suddenly frees you from much of that. It allows you to focus on the science, without any kind of barrier. That's been a really exciting process. To work openly feels a little bit dangerous–but you get a sense of freedom.

HASSAN: *Matt, thanks so much for a fascinating and inspiring conversation.*

MATT: No worries. It was nice to talk to you again.
